# Cholesterol-pyrene as a probe for cholesterol distribution on ordered and disordered membranes: Determination of spectral wavelengths

**DOI:** 10.1371/journal.pone.0201373

**Published:** 2018-08-10

**Authors:** Claudia Almeida, Anaëlle De Wreede, Antonin Lamazière, Jesus Ayala-Sanmartin

**Affiliations:** CNRS, Sorbonne Université, École normale supérieure, PSL University, INSERM, APHP,Hôpital Saint-Antoine, Laboratoire des biomolécules, LBM, Paris, France; Institut Curie, FRANCE

## Abstract

Biological membranes contain a large variety of lipids species compartmentalized in different domains heterogeneous in size, composition and dynamics. Cholesterol induces membrane ordered domains thanks to its affinity for saturated lipids. Membrane domains had been studied with fluorescent probes either linked to phospholipids and proteins or as individual fluorophore. However, no efficient formulation of a cholesterol probe has been available so far. Herein, we described a cholesterol-pyrene probe behaviour in heterogeneous membranes. We characterised the pyrene fluorescence spectra in liquid-ordered (Lo) and liquid-disordered (Ld) membranes. Using statistical multivariate analysis, we found out the most appropriate wavelengths for membrane domains studies. 373 nm and 379 nm were the most discriminant wavelengths to follow the liquid-ordered and the liquid-disordered environments. Cholesterol clustering behaviour was quantified by the modulation of the cholesterol-pyrene excimers peak (474 nm). In liquid-ordered membranes at low temperature, cholesterol-pyrene was found as multimers and as monomers. At high temperature, the liquid-ordered status of the membrane decreases and cholesterol-pyrene tends to cluster. In liquid-disordered membranes, cholesterol-pyrene was present mostly as monomers and the small quantity of excimers increased with temperature. Cholesterol-pyrene was used to test the ceramide effect on membranes, and presented a behaviour in agreement with the cholesterol behaviour reported in the literature. Overall, the presented data show that cholesterol-pyrene is an efficient sensor to study liquid ordered and liquid disordered organisation in membranes.

## Introduction

The cell membrane lipids are not randomly distributed but they form different membrane domains because of specific lipid-lipid and lipid-protein affinities. Membrane domains heterogeneity seems to have an important role in membrane organisation and in several cell functions as suggested by numerous experimental data (for reviews see [1–5]). The modulatory effects of cellular functions by lipid composition and membrane domain separation seem to be directly related to the physical-chemical properties of the different domains such as membrane thickness, lipid compaction and fluidity.

Among different membrane lipids, cholesterol is an important regulator and inducer of membrane domains separation. Its participation in the formation of the so called “raft domains” which were defined as cholesterol and sphingomyelin (SM) enriched membrane domains, is supported by different experimental data using membrane models as well as in cell membranes [6–10] and also by molecular dynamics simulations [11,12]. There is still controversy on the size of domains but some data suggest lipid immiscibility at nanometric scale and physiological temperatures [2,13]. The cholesterol ability to induce the formation of membrane domains is related to its stronger affinity for saturated lipid acyl chains compared to that for non-saturated chains [13–15] and by its ability to form an hydrogen bond with the amide group of sphingolipids [5]. It has been demonstrated that cholesterol has a tendency to be excluded from cis-unsaturated phosphatidylcholine (PC) and that its “ordering” effect is weaker compared to that with saturated PC [16,17]. The “rafts” are considered as liquid ordered domains (Lo) and the raft membrane bilayer is usually thicker than the surrounding liquid disordered domains (Ld). However, Cholesterol is not found exclusively in strong ordered domains but has also been observed in Ld domains all over the plasma membrane [5,18,19].

The ordered domains are not exclusively dependent on sphingomyelin and it has been reported a strong affinity of cholesterol for other lipids including PC containing saturated acyl chains [16] and ceramide (Cer). Ceramide is the product of the enzymatic cleavage of sphingomyelin by sphingomyelinase (SMase) which is secreted by the cells in pathologies involving apoptosis such as cancer. The substitution of sphingomyelin by ceramide in model membranes or by sphingomyelinase activity results in the modification of the Lo domains [20–22]. It had been proposed that ceramide and cholesterol compete for raft association mainly to the capacity of raft lipids with large head-groups to accommodate ceramide or cholesterol which have small head-groups [23].

The liquid ordered and liquid disordered membrane domains distributions have been studied by different approaches. ^2^H-labelled cholesterol had been used to study cholesterol distribution and its influence on membrane lipid distribution and domain formation by an NMR approach [24,25]. For a review of NMR approaches to study membrane domains see [26]. Other approaches including the use of fluorescent probes “specific” for different domains had been used. For example the membrane fluidity has been studied with environmental probes such as Laurdan [27–29], and Lo, Ld domains with fluorescent lipids (Di-Q, C6-NBD-SM, rho-DOPE) [8,30–32]. However, the specific cholesterol distribution by fluorescence has been less studied because of the lack of suitable and efficient cholesterol probes. Different studies showed that the performance of several fluorescent cholesterol derivatives were different depending on the experimental modalities. For example, TopFluor-cholesterol was more adapted than NBD-cholesterol derivatives to study the cholesterol partition in giant unilamellar vesicles (GUVs) by imagery, and a NBD-cholesterol derivative was more suitable in cellular trafficking studies [33,34]. To our knowledge, the cholesterol-pyrene probe described by Le Guyader and collaborators [35] is the less disturbing cholesterol-modified probe available. Similarly to the TopFluor-cholesterol, the pyrene group replaces the cholesterol “tail” with a similar size as the natural cholesterol and with the advantage that the pyrene is completely hydrophobic. Pyrene is a fluorescent probe that is sensitive to both, the environment (polarity, dielectric constant of the solvent, etc.), and to probe clustering. When two probes are spatially close enough (4–5 Å) [36], a fluorescence signal peak rises at about 475 nm (430–550 nm spectral contribution of dimeric-multimeric pyrene molecules; excimer signal). Thanks to this property, it is possible to observe changes in cholesterol-pyrene concentration and dilution. Therefore, the pyrene probe allows to follow changes of environment and molecular aggregation and it has also been successfully used to detect protein-protein interactions [37–39]. However, the pyrene fluorescence spectra is complex. The 360–430 nm range of the spectrum is considered as the contribution of the pyrene molecule in its monomeric state. There are at least 16 vibronic bands in the interval 371–397 nm [40] which result in at least five peaks in conventional spectra. Pyrene probe has been studied by several groups but led to inconsistent recommendations in terms of optimum wavelengths used as specific markers. 376 and 383 nm have been used for solvent or environment “polarity” [35,40]. Other wavelengths have been considered as “constant” or independent of the polarity of the environment such as 373, 376, 378, 388, 393 and 396 nm [35,39–42] and had been used for quantification of monomeric species. There is no obvious consensus due to the complex behaviour of the peaks intensities responding to the “polarity”, dielectric constant, dipole moment and geometries of the solvents and the environmental molecules in contact with pyrene and thermal agitation [40,43,44].

At low concentration cholesterol-pyrene (Py-met-chol) membrane behaviour is quite similar to cholesterol (Chol) [35] at least for simple membranes composed of a single phospholipid such pure DPPC, DOPC or in binary mixtures containing one single phospholipid and cholesterol. For example, the phase diagram of a DMPC cholesterol phase at 37°C shows that at 30% cholesterol, there is a transition from an Lo + Ld phase to the pure Lo phase. At higher cholesterol fraction the Lo phase is stabilized [45]. Cholesterol-pyrene can sense this transition because in DMPC, at 37°C, Le Guyader and collaborators [35] observed a net increase in cholesterol-pyrene aggregation (excimers) at 30% cholesterol that continue to increase when rising the cholesterol molar fraction. Considering this, and in order to study the cholesterol changes in heterogeneous membranes, we performed different spectral analyses on membranes in liquid ordered or in the liquid disordered states at different temperatures. We used lipids from biological sources with heterogeneous acyl chains, to best mimic the biological membranes. Instead of looking at changes in “polarity” by using spectral peak variations in intensity, we decided first to define the marker wavelengths corresponding specifically to the liquid ordered (Lo) or disordered (Ld) domains. In order to find out the Lo and Ld markers, we firstly performed a multivariate analysis using Ld (PC) and Lo (SM/Chol) membranes at different temperatures. Secondly, we analysed the behaviour of the ratios of these wavelengths and compared them to the previously suggested methods and the data from the literature. Thirdly, we compared the parameters of the Lo and Ld membranes using mixed membranes in which Lo and Ld domains coexist. Fourthly, we characterized the movement of the probe by anisotropy and finally, we studied the re-arrangements of cholesterol induced by the presence of ceramide in the membranes. The presented data showed that the Py-met-chol probe is an accurate molecule to study cholesterol clustering and distribution in lipid bilayers with the capacity to discern between Lo and Ld domains by using the recommended wavelengths.

## Materials and methods

### Materials

Egg yolk L-α-phosphatidylcholine (PC), egg sphingomyelin (SM) and cholesterol (Chol) were purchased from Sigma. C16-Ceramide (Cer) was from Enzo life sciences, pyrene-labelled cholesterol (Py-met-chol) was a kind gift of Dr. André Lopez (Toulouse, France). The mean acyl chain composition of eggPC is 33% of 16:0, 32% of 18:1, 17% of 18:2 and 12% of 18:0. For egg SM is 86% of 16:0, 6% of 18:0, 3% of 22:0 and 3% of 24:1.

### LUV preparation and incubation conditions

The appropriate amounts of lipids in a mixture of chloroform/methanol, 4/1 (v/v), were subjected to solvent evaporation. Large unilamellar vesicles (100 nm diameter LUVs) were prepared by extrusion in 0.5 mM HEPES buffer (pH 7.4) as described in [46]. The lipids were added at the desired proportions to obtain a suspension of 1 mg ml^-1^ LUVs in buffer. Py-met-chol was present at 1.8 or 3.6% lipid molar ratio.

After LUV preparation, the samples were kept at 4°C for several hours or overnight. To analyse the cholesterol changes depending on temperature we used two protocols. The heating protocol started from samples at 4°C, which were prepared in the quartz cell and measured in the fluorimeter from 10 to 55°C. Fluorescence spectra were recovered each 5°C step at a speed of about 1°C/min. In a first series of experiments, we rise the temperature to 70°C and observed that the excimers signal start to decrease after 55°C giving a bell shaped curve. We performed the experiments from 10 to 55°C because the higher temperatures are of no biological interest and the range 10–55°C seemed appropriate to characterize the cholesterol-pyrene behaviour in the membranes. For the cooling protocol, the samples were heated fast at 55°C (in about 5 minutes) and then cooled to 10°C in the fluorimeter. As for the heating protocol, spectra were recovered each 5°C step.

### Fluorescence

Pyrene fluorescence spectra acquisitions were performed with a Jasco fluorimeter as previously described [47]. Emission spectra were recorded from 360 to 600 nm using a 335 nm excitation wavelength. The excitation and emission band-pass were set at 5 nm and 2.5 nm respectively. 150 μl of buffer containing 2.5 μg of LUVs were added to a Hellma quartz cell. The temperature was regulated with a Peltier device. Spectra were obtained by duplicate and the buffer background was subtracted. The spectra were normalized by the integral to avoid artefacts due to thermal non radiative loss of excitation. Fluorescence anisotropy was characterized as previously described [48] by using the equation, r = (Ivv—G Ivh)/(Ivv + 2G Ivh) in which the Ivv and Ivh are the emission light intensities with the polarizers in different configurations (excitation-emission) and the G instrumental correction factor was defined as Ihv/Ihh and calculated for all wavelengths.

### Data analysis

The principal component analysis (PCA) of integral-normalized spectra treated as a multivariate (wavelength) set of data was performed with SIMCA software (Umetrics). The program find the components (correlated set of variables) that are responsible for the optimal separation of experimental observations (in our case spectra) in a multivariate space. Briefly, both, principal components analysis (PCA) and orthogonal partial least square-discriminant analysis (OPLS-DA) were performed. Different tools of the program SIMCA were used to evaluate the variables as markers. The component contribution of variables indicates the most relevant variables in each component. The variable importance of the projection summarises the importance of variables to explain X and correlate to Y in the projection plane. Biplot (scores vs loadings) displays similarities and dissimilarities between observations and allows interpreting observations in terms of variables. S-plots (covariance and correlation) and group-to-group comparisons were also performed. The univariate statistical treatment of experiments was performed with GraphPad Prism software. Graphs are given as means ± standard error.

## Results and discussion

The Py-met-chol probe has been studied in model and biological membranes [35]. It had been suggested that the environmental polarity should be characterized by changes in the 376 nm peak intensity, cholesterol-pyrene clustering by measuring the 475 excimers peak and to use the peak at 388 nm as a constant marker because in their conditions it depended only on probe concentration. However, studies of pyrene fluorescence by other authors were performed using different wavelengths. The reports suggest 376 or 378 nm as polarity markers. The wavelengths ranging from 450 to 488 were used for excimer contribution, 388 nm as constant and the range between 393 to 398 nm and 421 nm for “monomer” signal [35,38,39,41,42,49]. The range of the spectrum between 360 to 420 nm is specific of the molecular monomeric state and is composed of at least five principal peaks. It exhibits a complex behaviour in solvents with different polarity and different dielectric constants precluding a consensual hierarchical classification of peaks in function of “polarity”. Kalyanasundaram and collaborators [40] found at least 5 sensitive peaks which correspond to some of the 16 vibronic bands found in the 370 to 397 nm range of the spectrum. Moreover, fluorescence intensity changes due to thermal agitation are also different for several pyrene bands [44].

Taking into consideration this issues, and in order to study the Py-met-chol movement in a natural lipid environment, we decided to study the spectra in liquid ordered and liquid disordered membranes separately. We looked for the “marker” wavelengths able to differentiate both domains and the environmental independent constant point. We performed the experiments with eggPC (Ld) and eggSM/Chol (1/1) (Lo) membranes to tend toward biological membranes composition with heterogeneous acyl chains lipids (saturated and unsaturated). Although it generates complexity in term of effects, our goal is to understand the Py-met-chol behaviour in an environment with maximum possible similarities with biological conditions.

As recommended in [35], Py-met-chol was used at two concentrations below 5% (1.8 and 3.6% mol). The Ld membranes are more fluid and therefore more sensitive to temperature changes (ie temperature dependent membrane thickness variation) compared to the Lo membranes which are less fluid and show small fluctuations in membrane thickness throughout temperature variations [6,7,9]. Hence, to better understand Py-met-chol behaviour, the experiments were performed at different temperatures using specific heating and cooling protocols.

### Comparison of spectral behaviour after heating and cooling

To avoid analytical artefacts comparing the different experiments we normalized all the spectra by their integral. S1 Fig (supplementary information) shows the mean spectra from PC and SM/Chol LUVs at different temperatures after heating and cooling. For a specific temperature, the spectrum shape showed slight changes according to heating or cooling protocols. For example, the quantity of excimers (474 nm) at 10°C is higher before heating and lower after cooling. At 55°C, the excimer signal was lower after heating and higher before cooling. However, at 35°C the spectral difference between heating and cooling protocols were not significant.

In order to observe the differences between the Py-met-chol probe in Lo and Ld membranes, we performed spectra subtractions. This approach was not conclusive because we did not find a constant pattern for all tested conditions (S2 Fig). Therefore, to rationally find out the specific marker wavelengths (statistically significant) for Lo and Ld membranes for all the conditions tested, we performed a multivariate principal component analysis.

### Specific wavelengths for Lo and Ld membranes

In order to define specific wavelength markers for Lo and Ld membranes we compared the spectra of Py-met-chol in PC and SM/Chol (1/1) membranes at temperatures from 10 to 55°C by heating and cooling procedures. Py-met-chol was present at 1.8 or 3.6% mol. We compared four independent preparations of PC LUVs and five for SM/Chol LUVs containing Py-met-chol at 1.8% and seven independent preparations of PC LUVs and eight for SM/Chol LUVs containing Py-met-chol at 3.6% which result in 480 spectra, cooling and heating included. The variables are the spectral intensities of integral normalized wavelengths from 365 to 550 nm (186 variables). We performed both, principal components analysis (PCA) and orthogonal partial least square-discriminant analysis (OPLS-DA) and the analytical tools mentioned in the methods section.

**Fig 1:**
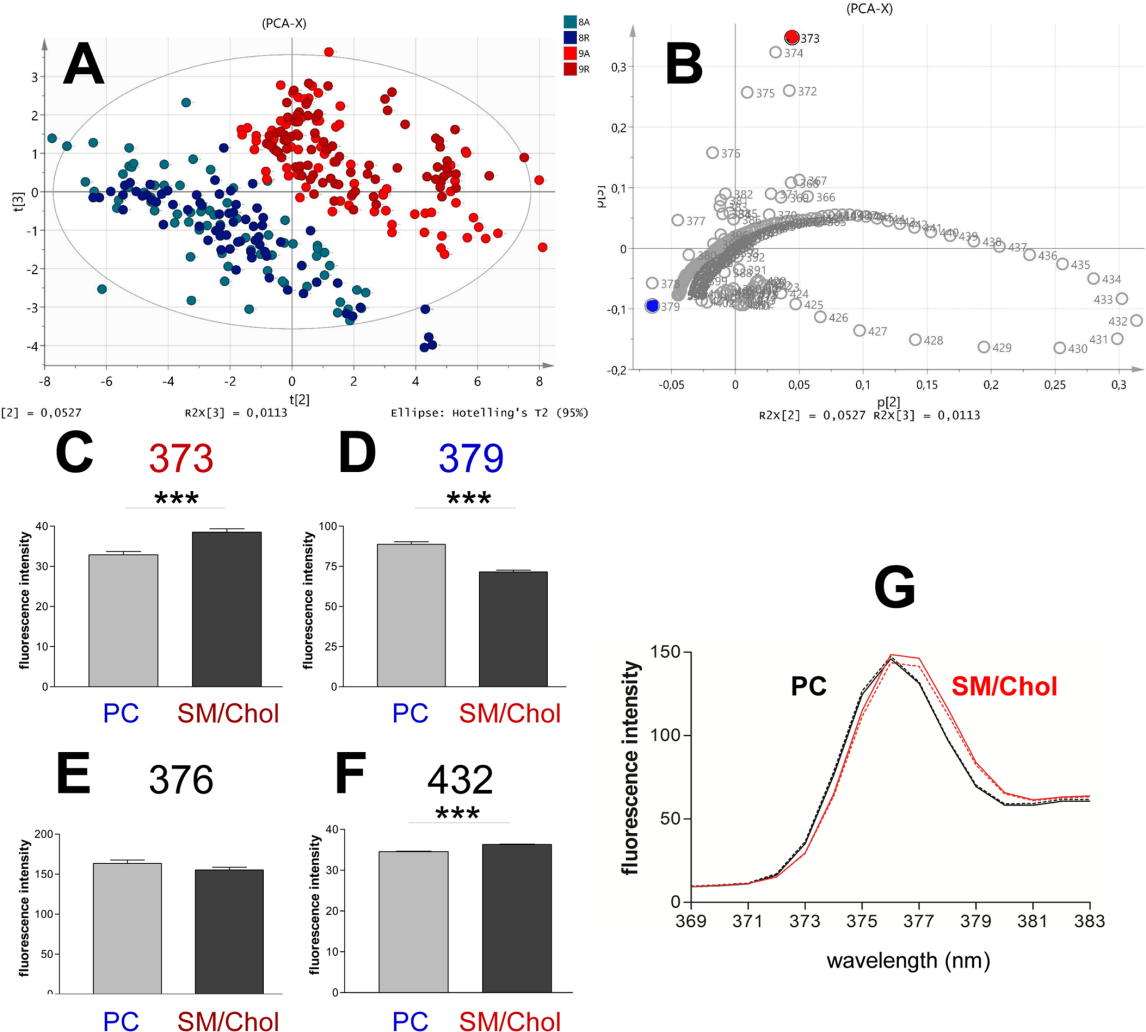
Principal component analysis of experimental individuals (spectra) and variables (wavelength). (A), Distribution of data in a plane corresponding to the second and third principal components (score scatter plot). Each point represents a spectrum from cooling and heating protocols (300 spectra). Points are, light blue PC during heating, dark blue PC during cooling, light red SM/Chol during heating and dark red SM/Chol during cooling. Notice the separation of PC and SM/Chol populations. (B), Correlation of variables (loading scatter plot). The strong anti-correlated variables in the y-axis direction are 379 nm (blue) and 373 nm (red). Quantification of fluorescence intensities at 373 (C), 379 (D), 376 (E) and 432 nm (F) for PC and SM/Chol spectra in (A). PC membranes N = 140, SM/Chol membranes N = 160. *** = P < 0.0001 by unpaired t-test. (G) Spectral peaks showing the red-shift when changing from a Lo to an Ld environment. PC membranes in black, SM/Chol in red, continuous lines for heating protocol and dotted lines for cooling. Mean spectra of 9 independent experiments.

We found out the variables (wavelengths) more accurate to differentiate between the Ld (PC) and Lo (SM/Chol) membranes. S3A Fig shows the score scatter plot for the two principal components of Py-met-chol (3.6%) spectra from PC and SM/Chol (cooling protocol) with no *a priori* consideration on data belonging to a particular population. The second component (Y-axis) clearly discriminated between the two membranes (PC vs SM/Chol), whereas the first component did not separate the two populations. This was explained by the fact that the temperature changes in membrane organisation has a high impact on the spectral shape (change in excimers/monomers ratio) as shown in S1 Fig. Fig 1A and 1B shows the score and loading representations for the second and third components. Two populations were clearly separated; PC bottom left quadrant and SM/Chol top right quadrant. For each membrane composition, the difference between cooling and heating protocols was not significant. Fig 1B corresponds to the correlation plane of variables for the components shown in Fig 1A. The variables (wavelengths) anti-correlated in the bottom left and top right quadrants were 379 nm and 373 nm respectively. It implicated that the population of PC correlated with an increase in 379 nm and a decrease in 373 nm. Inversely, the SM/Chol membranes showed an increase of 373 nm and a decrease in 379 nm. We also performed supervised orthogonal partial least square discriminant analysis in which the PC and SM/Chol membranes were considered as different classes. S3C and S3D Fig shows the scatter plot and the correlation plot which confirmed that the 373 and 379 nm variables were indeed strongly anti-correlated.

Therefore, we compared the fluorescence intensities of these wavelengths, the iso-emissive point 432 nm and the “polarity marker” 376 nm (Fig 1C–1F). The difference between the two populations (PC vs SM/Chol) was, statistically, very significant for the 373 and 379 nm wavelengths (P<0.0001). The 373 intensity values were 32.9 ± 0.8 for PC membranes and 38.5 ± 0.8 for SM/Chol. The 379 intensity values were 88.7 ± 1.6 for PC and 71.6 ± 1.1 for SM/Chol. The 376 peak intensity values were 163 ± 4 for PC and 155 ± 3 for SM/Chol membranes but with no statistical significance indicating that this peak was not suitable to differentiate between Lo and Ld membranes. The iso-emissive point was highly constant for each population (very small SEM) but the difference between the mean values (34.6 ± 0.1 for PC and 36.3 ± 0.1 for SM/Chol) was too small precluding its use as a marker. Thus, the strongest and highly significant difference between PC and SM/Chol membranes was accurately observed comparing 373 and 379 nm. These two wavelengths does not correspond to a conventional spectral peak but to the specific vibronic bands 372.51 and 378.99 nm [40]. The 376 nm peak previously used for “polarity” does not correspond to any vibronic band. Our analyses suggest that 373 and 379 nm wavelengths can be considered as appropriate candidate markers of Lo and Ld environments and were named cPyO3 and cPyD9 for **c**holesterol-**Py**rene L**O** environment marker (or L**D**), 37**3** and 37**9** nm respectively. Fig 1G shows the spectral peaks including the marker wavelengths in PC and SM/Chol membranes. Therefore, to validate their accuracy, we performed detailed comparisons for the Lo (SM/Chol) and Ld (PC) membranes. The wavelengths selected for the following analyses are listed in Table 1.

**Table 1:** Fluorescence wavelengths of interest for the study of membrane domains with Py-met-chol obtained by multivariate analysis and cited in the literature.

Wavelength (nm)	Interpretation	abbreviation
**373**	Liquid ordered (Lo) marker (this work)	cPyO3
**376**	“polarity” (from ref [35])	I1
**379**	Liquid disordered (Ld) marker (this work)	cPyD9
**388**	“constant” (from ref [35])	I3
**401**	“polarity” (this work)	pol
**432**	constant, iso-emissive point (this work)	iso
**474**	Py-met-chol multimerisation, excimers	exci

### Comparison between Lo and Ld membranes

**Fig 2:**
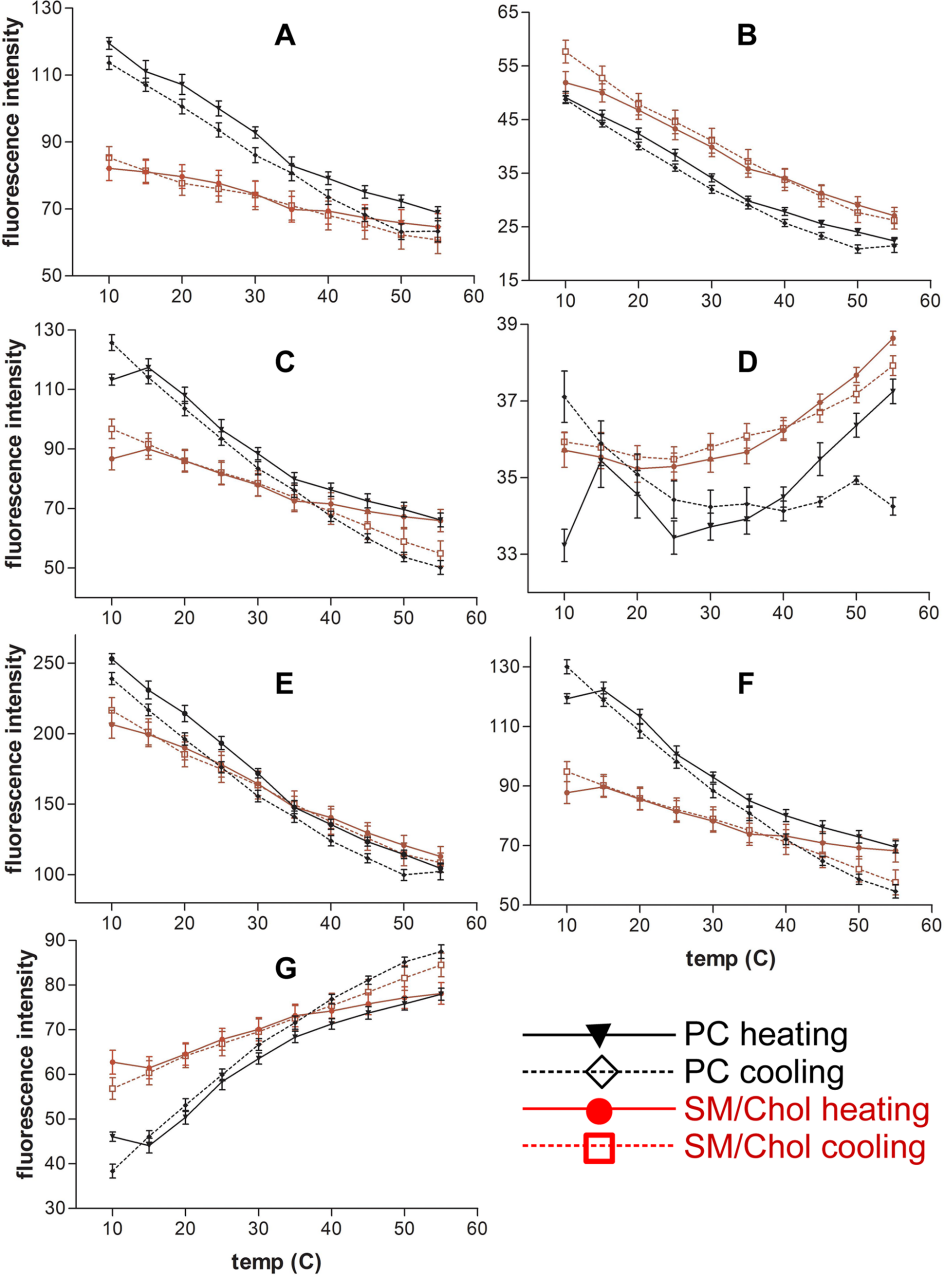
Evolution of Py-met-chol fluorescence intensities in function of temperature. The probe concentration in LUVs is 3.6%. (A), 379 nm cPyD9. (B), 373 nm cPyO3. (C), 388 nm, I3 “constant peak”. (D), 432 nm iso-emissive “constant point”. (E), 376 nm I1, “polarity peak”. (F), 401 nm “polarity”. (G), 474 nm excimer peak or multimer marker. Black lines PC LUVs, red lines SM/Chol. Continuous lines heating protocol and dotted lines cooling protocol. Means ± SEM of 7 independent experiments for PC and 8 for SM/Chol.

Fig 2 represents the temperature evolution of the band intensity corresponding to the relevant wavelengths for PC and SM/Chol LUVs in the presence of Py-met-chol at 3.6%. Similar behaviour was observed for the LUVs with 1.8% probe concentration (S4 Fig). For cPyD9 we observed that at low temperatures the values were high for PC and low for SM/Chol membranes (Fig 2A). However, the increase in temperature, which increases membrane fluidity, induced a decrease of the 379 nm band intensity. This paradoxical behaviour was due to the diminution of fluorescence in this zone of the spectra by the increase in excimer fluorescence intensity. The evolution of cPyO3 (Fig 2B) showed that the values for SM/Chol membranes were higher than for PC membranes at all temperatures in agreement with the fact that the Lo membranes are more stable over temperature than the Ld membranes.

For the previously considered constant peak which was suggested to depend only on probe concentration (388 nm), we observed that, at the same concentration and temperature, its behaviour was quite different in PC and SM/Chol LUVs indicating that it was also sensitive to other environmental factors such as polarity (Fig 2C). On the contrary, the iso-emissive point (432 nm) (Fig 2D) showed smaller variations in intensity and could be considered as the constant point of the spectra.

The previously reported polarity “marker” (376 nm, I1) (Fig 2E) was compared with 401 nm, a wavelength more sensitive to the environment (Fig 2F). We observed that 401 nm was able to distinguish the variation in “polarity” between PC and SM/Chol whereas 376 nm behaviour was similar for both membranes. The behaviour of 401 nm was close to that of 388 nm, a fact that questions the validity of 388 nm as a “constant” peak. Finally, the evolution of excimers (474 nm) (Fig 2G) showed that at low temperature the quantity of Py-met-chol multimers in SM/Chol membranes was higher than in PC membranes. The increase in temperature induced an increase in excimers for both LUVs. To avoid misinterpretations due to thermic and excimer driven fluorescence diminution and other artefacts we performed ratio-metric analyses of the data.

**Fig 3:**
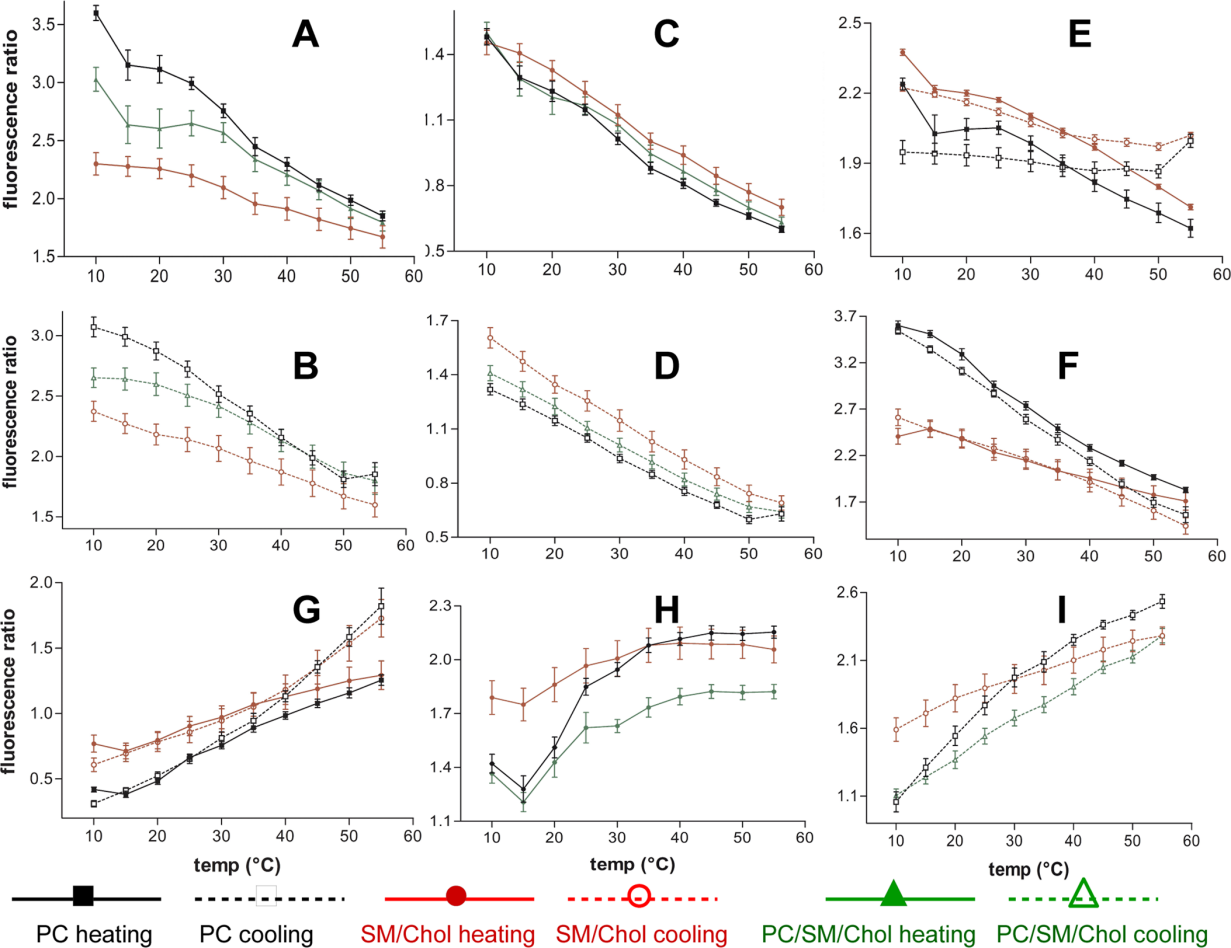
Ratios of Py-met-chol fluorescence wavelengths in function of temperature. The probe concentration in LUVs is 3.6%. (A,B), cPyD9 /iso. (C/D,), cPyO3 /iso. (E), I1/I3 (“polarity/constant”). (F), pol/iso. (G), exci/I3. (H,I), exci/iso. Black lines (■,□) PC LUVs, red lines (●,○) SM/Chol and green lines (▲,∆) PC/SM/Chol. Continuous lines heating protocol and dotted lines cooling protocol. Means ± SEM of 7 independent experiments for PC, 8 for SM/Chol and 6 for PC/SM/Chol.

As shown in Fig 3 (S5 Fig for Py-met-chol at 1.8% mol), the temperature evolution of cPyD9/iso and cPyO3/iso ratios were very similar to those of cPyO3 and cPyD9 (Fig 2). Strong differences were observed when we compared the I1/I3 which was supposed to characterize the evolution of polarity and pol/iso (Fig 3E and 3F and S5C and S5D Fig). In fact, the I3 peak was not exclusively dependent on the probe concentration and its intensity varied according to the environment. This could explain why the slope I1/I3 was smaller than that of pol/iso (401nm) which presented a behaviour closer to the curve cPyD9/iso. Therefore, we suggest that the iso-emissive correction is more adequate to represent a constant concentration-independent spectral point. The concept of polarity in the membrane bilayer was quite difficult to interpret but “polarity” seemed to be represented by the cPyD9/iso ratio (Fig 3A and 3B). When comparing the exci/I3 (Fig 3G and S5E Fig) and exci/iso ratios (Fig 3H and 3I and S5F Fig) the iso-emissive point was more accurate to follow probe clustering. Using the exci/I3 parameter the excimer formation increased continuously when temperature rose up. On the contrary, the exci/iso ratio showed that the excimer formation reached a plateau, consistent with what expected from membrane properties. Overall, the data suggested that to characterize py-met-chol in the membrane domains, a close look at the evolution of cPyO3/iso, cPyD9/iso and exci/iso markers is more accurate.

Therefore, to evaluate accurately the evolution of cholesterol-pyrene in membranes we looked at two principal parameters; the cPyO3/cPyD9 ratio which indicated the proportions of the two domains in the membranes, and the exci/iso ratio that indicated the degree of cholesterol-pyrene association or dissociation. The behaviour of excimer formation (Fig 3H and 3I and S5F Fig) showed that at low temperature the quantity of excimers is higher in SM/Chol than in PC LUVs. After temperature increase, the amount of excimers in SM/Chol increased but in a small proportion (about 15–20%). This could be due to a Cholesterol-pyrene movement from a membrane zone acquiring a temperature induced Ld character to a membrane zone of a strong Lo character. However, we cannot exclude the possibility that the excimers increase is also due to an increase in thermic driven molecular encounters. Overall, the data was consistent with the fact that the SM/Chol membranes show less temperature sensitivity compared to the PC membranes. The increase of excimers for the PC membranes is stronger, from 60% at 3.6% to about 100% (two fold) at 1.8% of the probe. This suggests that the increase in temperature provoked the disappearance of a proportion of “weak” Lo domains with the concomitant concentration of cholesterol in the low proportion of resting Lo domains enriched in saturated acyl chains. It is important to note that the cooling and heating protocols showed different behaviours. The amount of excimers was higher at the beginning of the cooling protocol compared to the amount at 55°C after heating protocol. With gradual heating, the preformed domains at 4°C underwent lipid reorganisation progressively. For the cooling protocol, the samples were rapidly heated to 55°C and therefore the lipids had a short time to reorganise. This led to a brutal domains perturbation with a small proportion of remaining Lo domains. Thus, cholesterol could move more freely or accumulate in these domains with, as consequence, more excimers at 55°C before the cooling protocol. Thus, it is evident that with the heating protocol, the excimers formation was not due only to thermic movements but by local cholesterol concentration.

**Fig 4:**
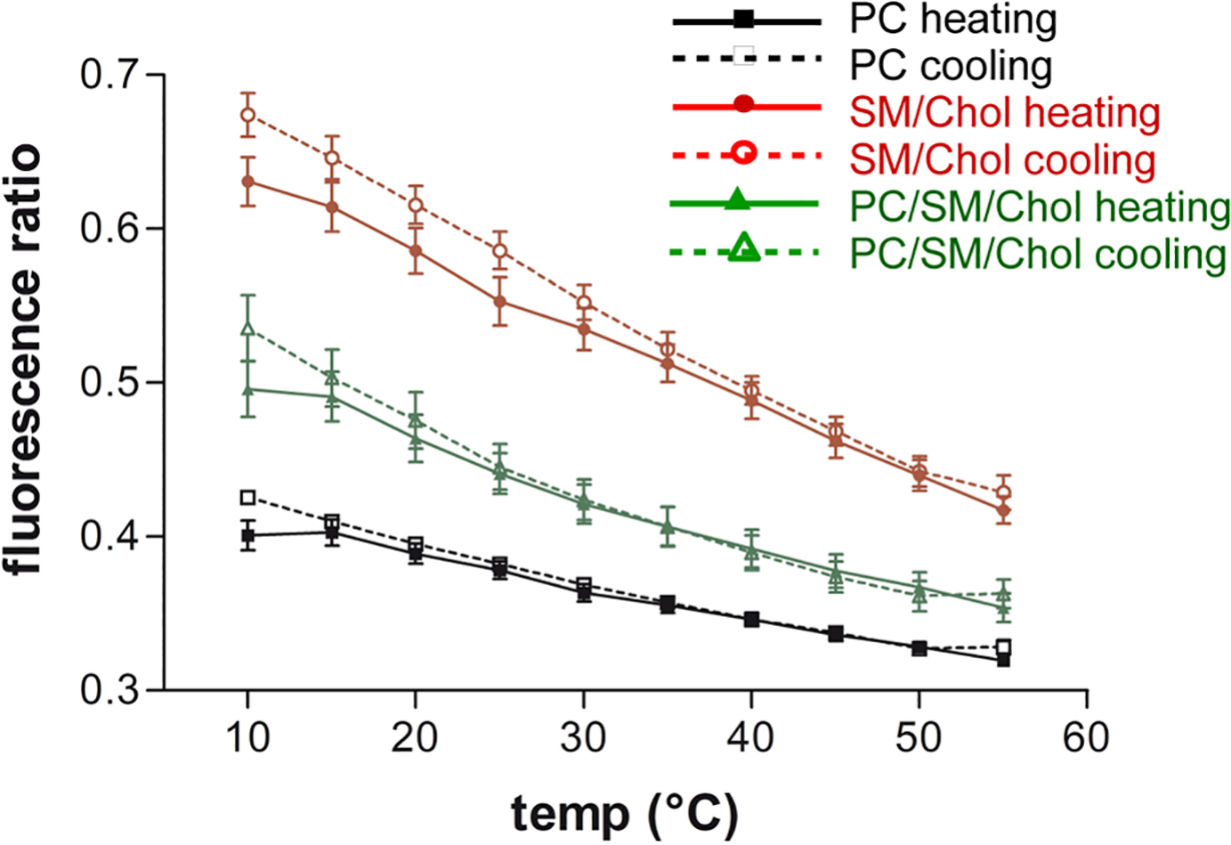
cPyO3/cPyD9 ratios of Py-met-chol fluorescence as function of temperature. The probe concentration in LUVs is 3.6 in. Black lines (■,□) PC LUVs, red lines (●,○) SM/Chol and green lines (▲,∆) PC/SM/Chol. Continuous lines heating protocol and dotted lines cooling protocol. Means ± SEM of 7 independent experiments for PC, 8 for SM/Chol and 6 for PC/SM/Chol.

Fig 4 shows the cPyO3/cPyD9 ratio for both PC and SM/Chol membranes and rise several points (S6 Fig for cholesterol-pyrene at 1.8%). Firstly, the heating and cooling protocols revealed almost the same behaviour. The observed difference between heating and cooling protocols were small for SM/Chol membranes at low temperatures. Secondly, the effect due to temperature change for PC membranes showed a low slope compared to the SM/Chol. Considering that the change in cPyO3 was quite similar for both membranes (Fig 3C and 3D), the slope difference could be attributed to the cPyD9 contribution which was more sensitive to temperature for PC than for SM/Chol membranes (Fig 3A and 3B). Finally, the error bars for this ratio were very small indicating that this ratio was, statistically, an accurate parameter for membrane domains characterisation. It is interesting to note that the 373 and 379 bands formed part of the ascending and descending sides of the peak centred at 376 nm analogously to the large spectrum of Laurdan in which the gel and liquid fluid markers were found at 440 and 490 nm (Fig 1G).

### Anisotropy

**Fig 5:**
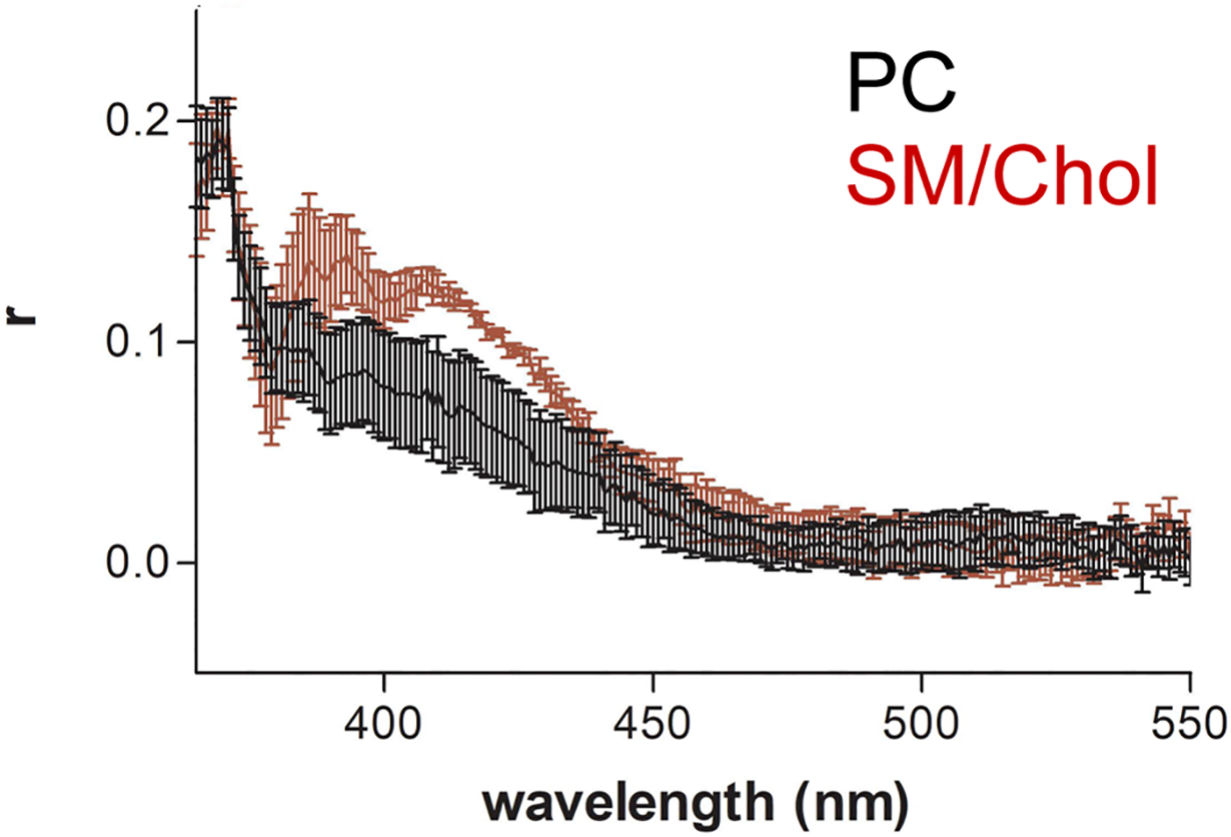
Fluorescence anisotropy (r) of Py-met-chol. LUVs were incubated at 35°C. Py-met-chol is at 3.6%, PC LUVs in black and SM/Chol in red. Curves are the mean ± SEM of four independent experiments.

In order to characterize the Py-met-chol movement in the different domains we performed anisotropy analyses. Fig 5 shows the anisotropy of Py-met-chol in PC and SM/Chol LUVs at 35°C. The graphics for temperatures 15 and 50°C are shown in S7 Fig. The right part of the spectrum (440–550 nm) corresponds to the excimer signal. We observed no clear difference between the PC and SM/Chol membranes. This fact indicates that the Py-met-chol found in cholesterol rich domains (Lo character) presented similar degree of movement in PC and SM/Chol and this for all temperatures. The left part of the graphics (365–430 nm) corresponds to the contribution of monomer signal. It was clear that for all temperatures the anisotropy values were higher for the SM/Chol LUVs compared to PC membranes. This could be due to the fact that the cholesterol is able to make a hydrogen bond with SM. Overall, this reveals that in the monomeric state, Py-met-chol environment in SM/Chol was more “rigid” than in PC. These results are in agreement with the properties of cholesterol in SM/Chol versus PC membranes and indicates the existence of different types of domains with low content of cholesterol in membranes with different acyl chains.

Two questions rise from the anisotropy data: why multimers show less anisotropy than monomers? And why the excimers anisotropy is similar for SM/Chol and PC? Both questions can be answered by the following argument. In fact, the monomers are found between phospholipids and the multimers between phospholipids and other cholesterol molecules. The cholesterol molecule occupy less space than the long acyl chains of phospholipids that are spread and then, the pyrene moiety, which is found close to the centre of the bilayer have more space to move. In other words cholesterol dimerization increases more the space in the bilayer centre than the monomeric cholesterol that present a movement restrained by the long acyl chains of phospholipids. This effect is similar to the known “paradoxical” fact that cholesterol increase the disorder of a membrane in a gel phase.

### Cholesterol distribution in Ld-Lo mixed membranes

To study in further details the behaviour of the probe in membranes with both Lo and Ld domains, we performed experiments with PC/SM/Chol (1/1/1) LUVs. As shown in Fig 3A and 3B and S8A and S8B Fig, at 10°C the value of cPyD9/iso ratio of PC/SM/Chol membranes was intermediate between PC and SM/Chol. This is consistent with the coexistence of two different domains in which cholesterol was present. The difference between the three LUVs was maintained with the increase in temperature to 25–30°C. However, at higher temperatures (35–55°C), the cPyD9/iso values for PC/SM/Chol LUVs were similar to the PC membranes indicating higher global membrane fluidity around the monomeric cholesterol-pyrene for PC/SM/Chol membranes compared to SM/Chol. The cooling and heating protocols showed similar behaviour. The temperature evolution of cPyO3/iso showed that PC/SM/Chol membrane values were intermediate between the PC and SM/Chol for all temperatures (Fig 3C and 3D and S8C and S8D Fig). The evolution of the cPyO3/cPyD9 ratio exhibited a clear separation between the three membranes for all temperatures and for both protocols (Fig 4 and S6 Fig) indicating the existence of zones with Lo character in the PC/SM/Chol LUVs at all temperatures.

The excimer formation in PC/SM/Chol membranes was also intermediate compared to PC and SM/Chol membranes. At 10°C the exci/iso ratio was similar to the PC membranes but with the increase of temperature, there was a lowering in the excimers increase similarly to SM/Chol membranes (Fig 3H and S8E Fig). For the cooling protocol, when the membranes were quickly heated to 55°C, the excimer ratio for PC/SM/Chol was similar to SM/Chol membranes. Cooling the sample provoked a difference between membranes for the excimers ratio and PC/SM/Chol membranes became more similar to PC membranes at 10°C (Fig 3I and S8F Fig). In summary, cholesterol-pyrene excimers formation for PC/SM/Chol membranes was similar to PC membranes at lower temperatures and similar to SM/Chol membranes at higher temperatures. In accordance with the fact that the Lo domains are less sensitive to the temperature compared to the Ld domains we can deduce that during heating, cholesterol-pyrene had a tendency to move to the Lo domains. These temperature effects probably induced a change in the equilibrium between micro- and nanodomains [11,50]. In these mixed membranes, at high temperatures, the dimers would be in Lo zones and the monomers in the Ld regions, a situation that also explain the observed behaviour of the cPyO3/cPyD9 ratio values from monomers.

### Displacement of cholesterol by ceramide

**Fig 6:**
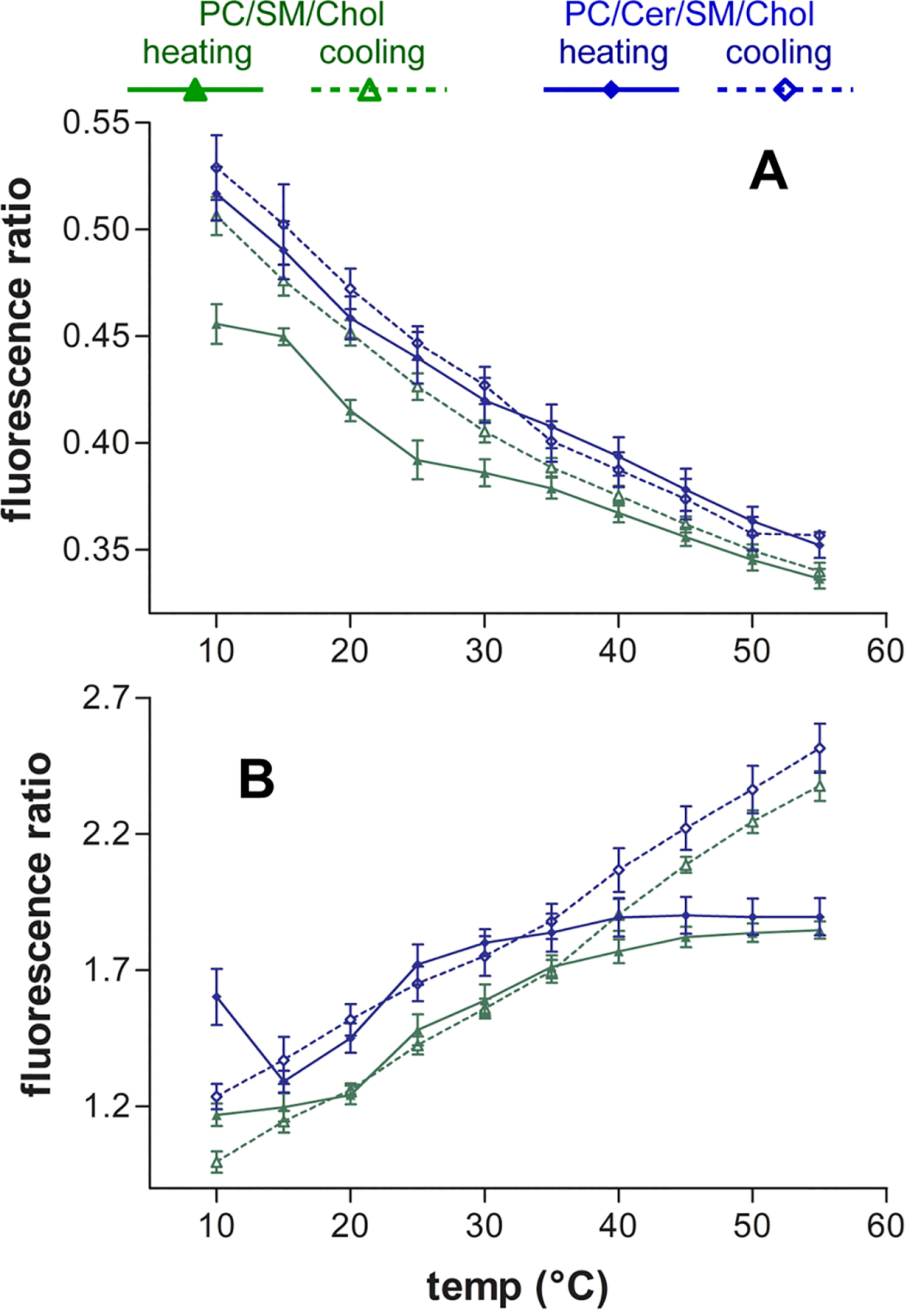
Ceramide effect on the evolution of Py-met-chol fluorescence in function of temperature. The probe concentration in LUVs is 3.6%. (A), cPyO3/cPyD9 ratio. (B), exci/iso ratio. Green lines (▲,∆) PC/SM/Chol LUVs, blue lines (♦,◊) PC/SM/Cer/Chol LUVs. Continuous lines heating protocol and dotted lines cooling protocol. Means ± SEM of 3 independent experiments.

It was reported that the conversion of sphingomyelin into ceramide induces a reorganisation of the membrane, which results from the competition of cholesterol and ceramide for sphingomyelin. Therefore, we tested whether the Py-met-chol probe was able to detect this reorganization. Megha and London [23] studied this phenomenon after TNF stimulation. The sphingomyelinase changes 80% of the Plasma membrane SM into ceramide. Taniguchi and collaborators [22] reported a conversion of 36% of SM into Ceramide, but considering that there is some pool of intracellular SM non exposed to the sphingomyelinase, they concluded that the quantity of ceramide at the plasma membrane must be higher. In PC/SM/Chol membrane models, Silva et al [21] reported a conversion of 50% of SM into ceramide. Considering these results, we performed experiments with PC/SM/Cer/Chol (1/0.5/0.5/1) membranes to mimic sphingomyelinase standard action. S9 Fig shows that the cPyD9/iso and cPyO3/iso values were smaller compared to the LUVs without Cer (PC/SM/Chol) indicating changes in cholesterol-pyrene environment. For cPyO3/cPyD9 ratio (Fig 6), we observed higher values for all temperatures especially for heating protocol. Hence, in the presence of ceramide, the monomeric cholesterol-pyrene “moves” to an environment of higher Lo character. These results were in agreement with the fact that ceramide provokes the stabilization of rafts [23], and the formation of gel domains [20,21,32]. Simultaneously, the evolution of the excimer formation (Fig 6) showed that cholesterol-pyrene in the presence of ceramide increased its tendency to aggregate, confirming that the presence of ceramide in this membrane induced a rearrangement of cholesterol. However, it is difficult to deduce the exact distribution of cholesterol in such complex membranes. Staneva et al had been shown that a PC/SM/Cer/Chol membrane can be composed of 6 different lamellar phases at 22 and 38°C [51].

## Conclusions

**Fig 7:**
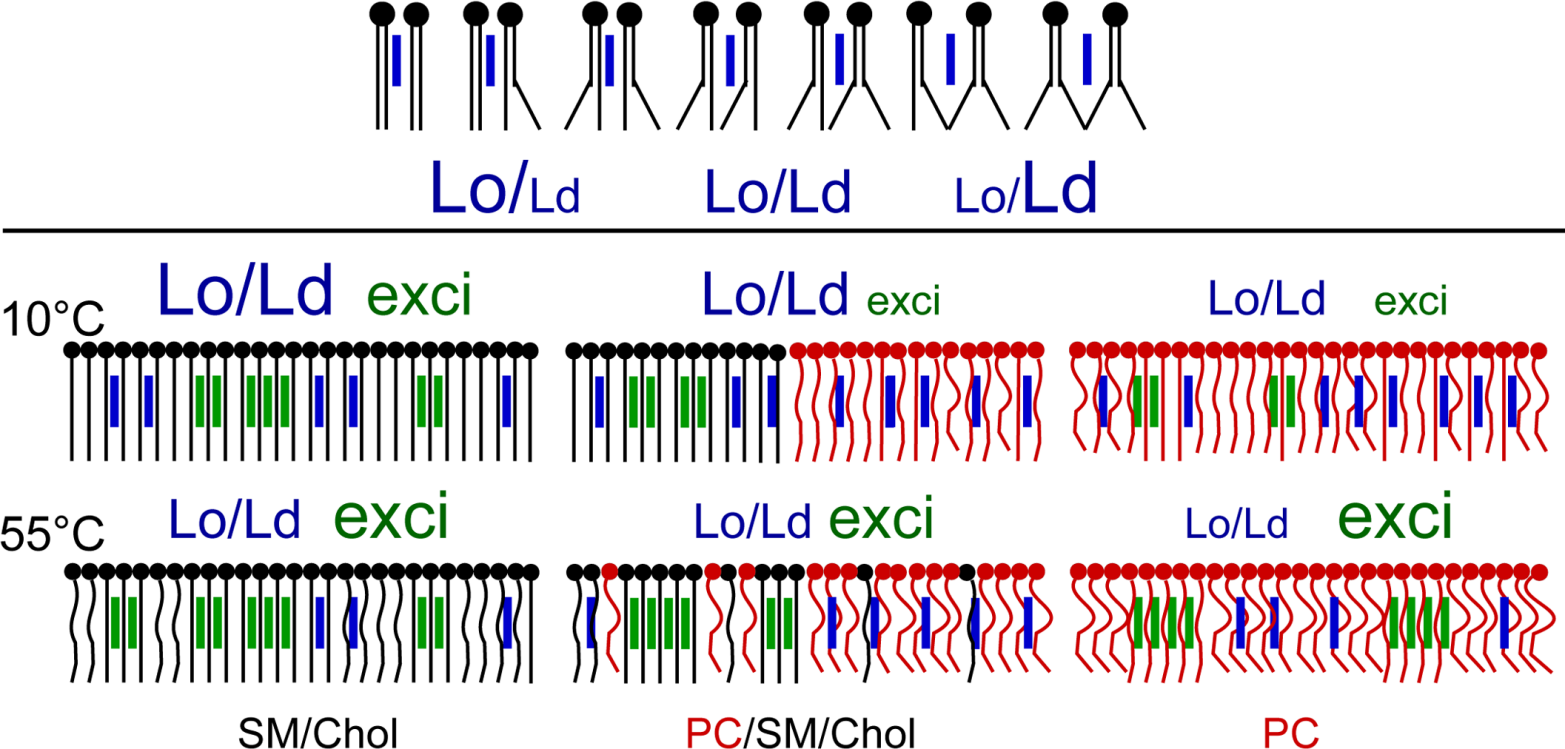
Schematic interpretations of cholesterol-pyrene probe behaviour in different membranes. Top, schematic representation of the changes in Lo/Ld character ratio depending on the saturation of phospholipids surrounding the cholesterol-pyrene. See text for details. Bottom, phospholipids and sphingolipids are represented by round tailed figures and cholesterol by vertical bars. The different forms of tails represent the acyl chains, which have different mobility and degree of unsaturation. SM black, PC red, multimeric cholesterol-probe green and monomeric form blue. In top, the membranes at low temperature and in the bottom at high temperature. The size of letters for Lo, Ld and exci correspond to the observed relative experimental values. Lo/Ld ratio mainly for the monomeric cholesterol. Exci is the exci/iso ratio characterising cholesterol clustering. See the text for detailed explanation.

Membrane domains had been studied with the help of numerous fluorescent probes. However, the specific cholesterol distribution had been less studied due to the lack of efficient cholesterol probes. In this work, we explored the abilities of a cholesterol-pyrene to sense ordered and disordered domains in lipid membranes. We characterised the pyrene fluorescent spectrum by using a statistical principal component analyses. We defined in a wide range of temperatures, the most accurate wavelengths associated with liquid ordered (Lo) and liquid disordered (Ld) membrane domains, respectively at 373 nm and 379 nm (cPyO3 and cPyD9). These wavelengths form part of a fluorescent peak (Fig 1G) that, as for Laurdan and other environment sensitive probes, experimented a red-shift in a more “fluid” environment. We observed that the 373/379 ratio changed depending of the environment of the pyrene. The top part of Fig 7 illustrate the influence of the phospholipids on the 373/379 ratio. The more the acyl chains surrounding the Cholesterol-pyrene are saturated, the higher the 373/379 ratio is, and on the contrary, the more unsaturated they are, the smaller the ratio is. These two markers are part of the pyrene monomeric contribution of the spectrum. The third marker used for cholesterol aggregation was the well-known excimers peak (474 nm). This marker signal came essentially from Lo domains, which were enriched in cholesterol. Finally, to reduce the experimental variations, we recommended the use of the iso-emissive point (432 nm) as a constant reference spectral point.

By using the 373/379 and 474/432 ratios we observed that the kinetics of domains dynamics were slightly different when heating or cooling the membranes. The behaviour of cholesterol-pyrene in different membranes used in this study is illustrated in bottom part of Fig 7. In cold Lo membranes (SM/Chol) in which the ordered domains are larger, cholesterol-pyrene molecules were found as multimers or monomers because cholesterol-pyrene displaced between the numerous saturated acyl chains of lipids and cholesterol. When temperature increased, the acyl chains “melting” reduced the Lo behaviour of the membrane and cholesterol-pyrene tended to accumulate in saturated lipid rich zones then increasing the excimers amount. This implied a moderate reduction in the proportion of the Lo domains, which was expected by a rise in temperature.

In cold Ld membranes such as PC with heterogeneous acyl chains, cholesterol-pyrene was present mostly as monomers but some excimers were present corresponding to small ordered domains composed of cholesterol and lipids containing saturated acyl chains. Rising the temperature provokes the accumulation of cholesterol-pyrene in still ordered zones enriched with saturated phospholipids.

In mixed membranes, cholesterol-pyrene showed intermediate behaviour. At low temperatures, it had tendency to associate with the saturated lipids of the Lo domains but was also present in the disordered domains as shown by its intermediate spectral behaviour. Rising the temperature provoked the increase of cholesterol-pyrene clustering probably by the association of cholesterol-pyrene with Lo zones present in the membrane.

The capacity of Py-met-chol to be used as a cholesterol probe was also tested by studying the ceramide effect on membranes, which showed that ceramide increases the Lo character of the membrane.

In conclusion, for interpretation of fluorescence data in membranes with constant cholesterol content and with Py-met-chol at about 2–3% mol, we suggest to look at the 373/432 and 379/432 ratios to characterize the monomeric cholesterol-pyrene environmental changes. For the two other parameters 474/432 (excimers) and 373/379 (Lo/Ld global contribution) we suggest that, at constant temperature and for a membrane enriched in saturated acyl chains and cholesterol, the increase in 474/432 ratio indicates the concentration of the probe and therefore a reduction in the proportion of Lo character and inversely a ratio decrease indicates an increase in Lo proportion in which the probe is diluted. For a membrane enriched in unsaturated acyl chains and poor in cholesterol, the changes in 474/432 ratio revealed the concentration-dilution of cholesterol-pyrene and therefore the dynamics of Lo zones in the membrane. For the 373/379 ratio, which indicates the movement of monomeric cholesterol-pyrene, the interpretation must take into account the excimers dynamics. The changes in this ratio indicate the global changes of the probe into Lo and Ld environments. It must be considered that in SM/Chol enriched membranes the cholesterol-pyrene could be present as monomers surrounded by cholesterol. As a whole, our experiments and interpretations are in accordance with the available data on the literature suggesting that the Py-met-chol, at low concentrations, is an adequate cholesterol probe for the study of cholesterol dynamics in heterogeneous membranes at temperatures in the range of living organisms.

## Supporting information

S1 Fig Mean spectra of PC and SM/Chol LUVs at different temperatures after cooling and heating. (A) spectra at 10°C, (B) at 35°C and (C) at 55°C. Red lines PC LUVs, black lines SM/Chol LUVs. Continuous lines from heating protocol and dotted lines from cooling protocol. Each line is the mean of 7 (PC) and 8 (SM/Chol) spectra from independent experiments.
(JPG)

S2 Fig Subtraction of Ld-Lo Py-met-chol spectra (3.6%) at different temperatures. PC mean spectrum minus SM/Chol mean spectrum at (A) 10°C, (B) 35°C and (C) 55°C. Continuous lines for heating protocol and dotted line for cooling protocol. Mean of 7 independent experiments for PC and 8 for SM/Chol.
(JPG)

S3 Fig Multivariate analysis of experimental individuals (spectra) and variables (wavelength). (A, B) Principal component analysis. (A), Distribution of data in a plane of axis corresponding to the two principal components (score scatter plot). Each point represents a spectrum from cooling and heating protocols (300 spectra). In dark blue points PC during heating, light blue PC during cooling, dark red SM/Chol during heating and light red SM/Chol during cooling. Notice the separation of data in two populations (PC and SM/Chol) along the y-axis (second component). The first component (x-axis) is related to the strong spectral changes due to the temperature-dependent formation of excimers. (B), Correlation of variables (loading scatter plot). The strong anti-correlated variables in the x-axis direction are the wavelengths from 433 to 550 nm corresponding to the excimer signal at the left and the wavelength from 365 to 431 nm corresponding to the monomeric signature at right. 373 nm (red circle), 379 nm and 432 nm (blue circle) are points of interest separated in the y-axis direction (see text). (C,D) Orthogonal partial least squares discriminant analysis (OPLS-DA) considering PC and SM/Chol membranes as different classes. (C) Score scatter plot showing the variability within classes (due mostly to temperature). Each point represents a spectrum from cooling protocol (150 spectra). Blue points PC, red SM/Chol. (D), Correlation of variables (loading scatter plot). The strong anti-correlated variables thus, with the higher discriminatory power between the classes are 373 nm and 379 nm.
(JPG)

S4 Fig Evolution of Py-met-chol fluorescence intensities in function of temperature. The probe concentration in LUVs is 1.8%. (A), 379 nm Ld marker (cPyD9). (B), 373 nm Lo marker (cPyO3). (C), 388 nm I3 “constant pic”. (D), 432 nm iso-emissive “constant point”. (E), 376 nm I1 “polarity pic”. (F), 401 nm “polarity”. (G), 474 nm excimer pic or multimer marker. Black lines PC LUVs, red lines SM/Chol. Continuous lines heating protocol and dotted lines cooling protocol. Means ± SEM of 4 independent experiments for PC and 5 for SM/Chol.
(JPG)

S5 Fig Ratios of Py-met-chol fluorescence wavelengths in function of temperature. The probe concentration in LUVs is 1.8%. (A), cPyD9/iso. (B), cPyO3/iso. (C), I1/I3 (“polarity/constant”). (D), pol/iso. (E), exci/I3. (F), exci/iso. Black lines PC LUVs, red lines SM/Chol. Continuous lines heating protocol and dotted lines cooling protocol. Means ± SEM of 4 independent experiments for PC and 5 for SM/Chol.
(JPG)

S6 Fig cPyO3/cPyD9 ratios of Py-met-chol fluorescence as function of temperature. The probe concentration in LUVs is 1.8%. Black lines PC LUVs, red lines SM/Chol and green lines PC/SM/Chol. Continuous lines heating protocol and dotted lines cooling protocol. Means ± SEM of 4 independent experiments for PC, 5 for SM/Chol, and 6 for PC/SM/Chol.
(JPG)

S7 Fig Fluorescence anisotropy (r) of Py-met-chol. LUVs were incubated at (A) 15°C, (B) 50°C. Py-met-chol is at 3.6%, PC LUVs in black and SM/Chol in red. Curves are the mean ± SEM of four independent experiments.
(JPG)

S8 Fig Ratios of Py-met-chol fluorescence in mixed membranes. The probe concentration in LUVs is 1.8%. (A), cPyD9/iso during heating. (B), cPyD9/iso during cooling. (C), cPyO3/iso during heating. (D), cPyO3/iso during cooling. (E),exci/iso during heating. (F), exci/iso during cooling. Black lines PC LUVs, red lines SM/Chol and green lines PC/SM/Chol. Continuous lines heating protocol and dotted lines cooling protocol. Means ± SEM of 4 independent experiments for PC, 5 for SM/Chol and 6 for PC/SM/Chol.
(JPG)

S9 Fig Ceramide effect on the evolution of Py-met-chol fluorescence in function of temperature. The probe concentration in LUVs is 3.6%. (A), cPyD9 marker. (B), cPyO3 marker. Green lines (▲,∆) PC/SM/Chol LUVs, blue lines (♦,◊) PC/SM/Cer/Chol LUVs. Continuous lines heating protocol and dotted lines cooling protocol. Means ± SEM of 3 independent experiments.
(JPG)
