# Supplementary figures and images for: Cholesterol-pyrene as a probe for cholesterol distribution on ordered and disordered membranes: Determination of spectral wavelengths
Source: PLoS One. 2018 Aug 10;13(8):e0201373. doi: 10.1371/journal.pone.0201373 (PMC6086420; doi:10.1371/journal.pone.0201373)

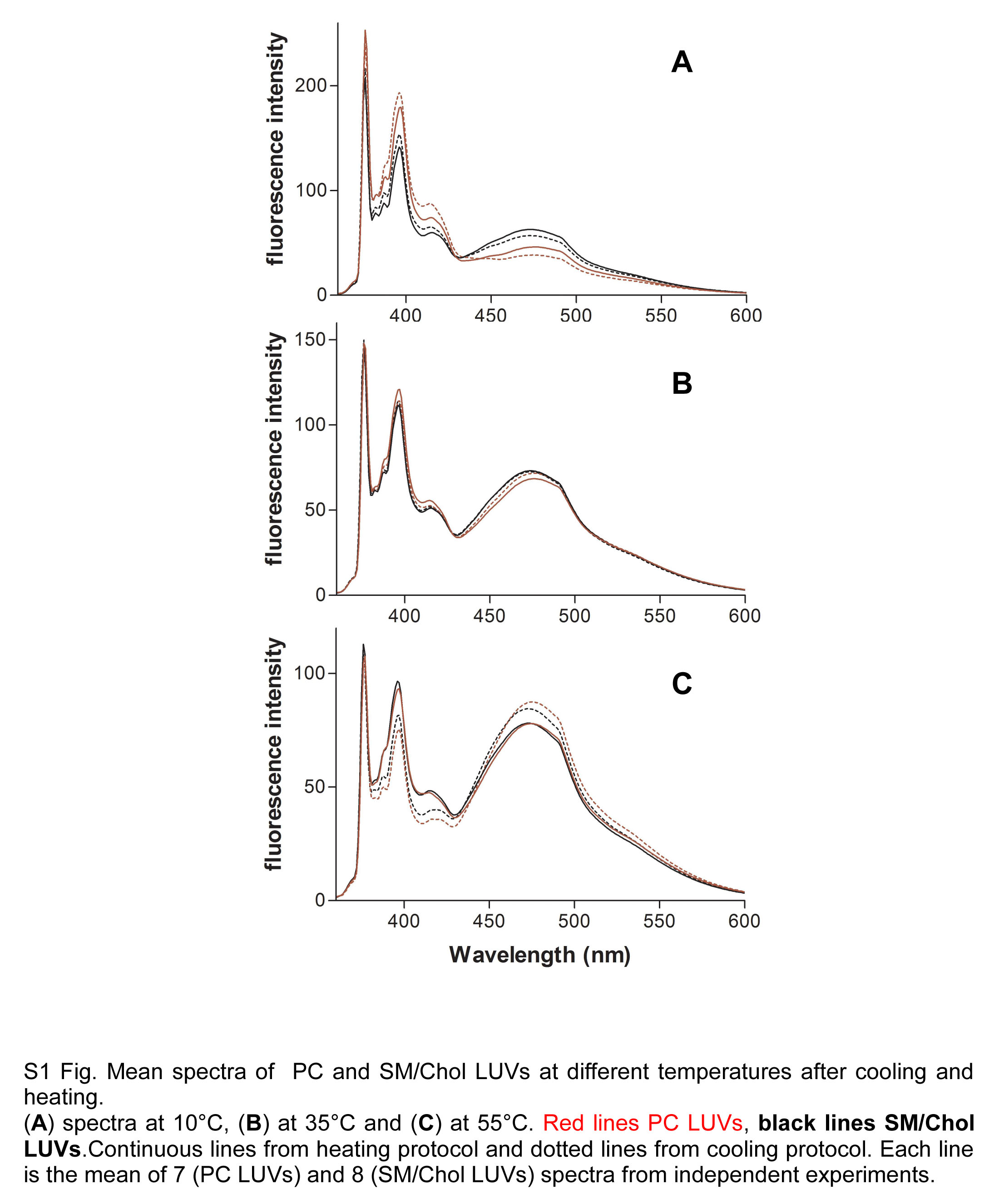

Supplement: S1 Fig — (A) spectra at 10°C, (B) at 35°C and (C) at 55°C. Red lines PC LUVs, black lines SM/Chol LUVs. Continuous lines from heating protocol and dotted lines from cooling protocol. Each line is the mean of 7 (PC) and 8 (SM/Chol) spectra from independent experiments. (JPG) [file pone.0201373.s001.jpg]

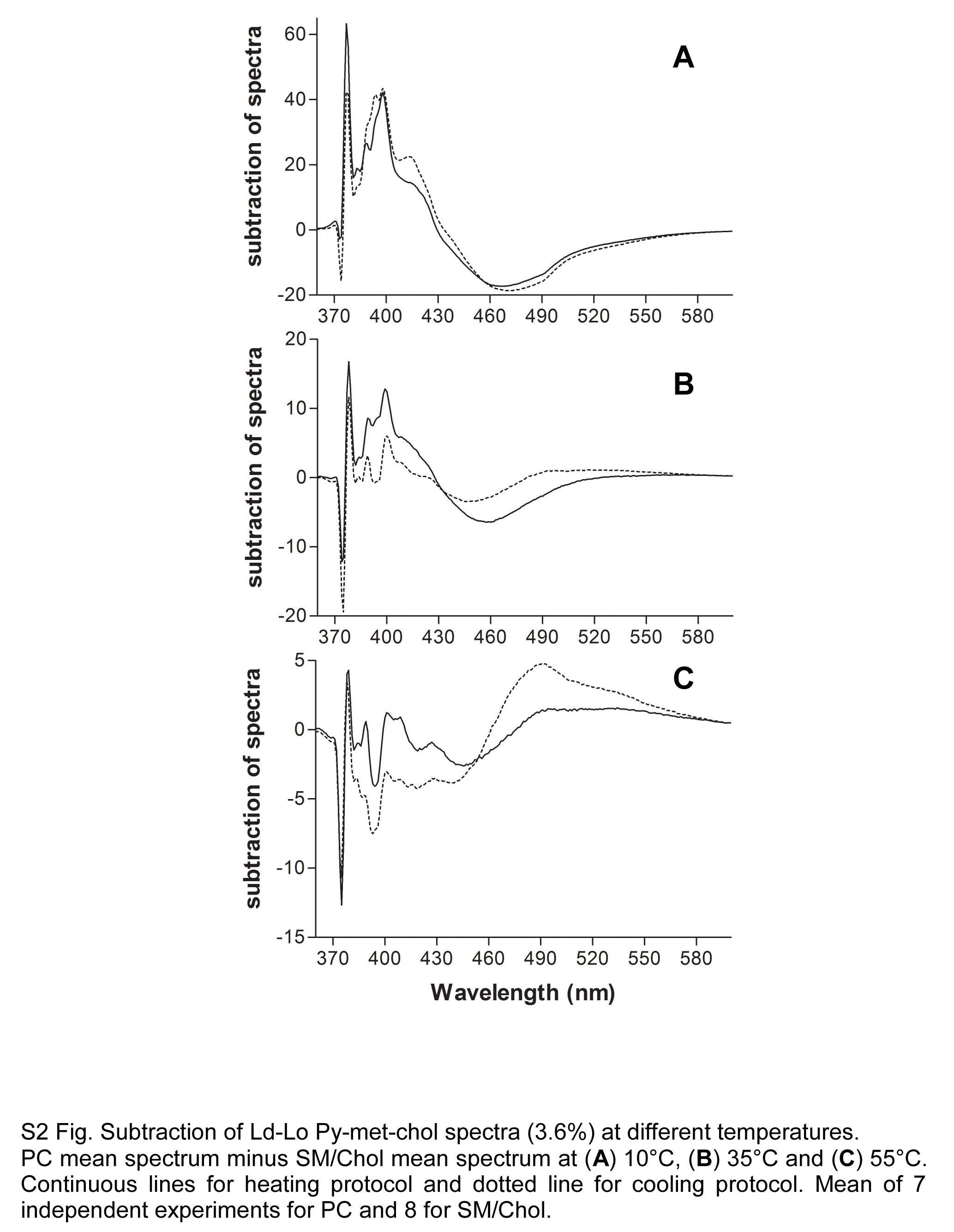

Supplement: S2 Fig — PC mean spectrum minus SM/Chol mean spectrum at (A) 10°C, (B) 35°C and (C) 55°C. Continuous lines for heating protocol and dotted line for cooling protocol. Mean of 7 independent experiments for PC and 8 for SM/Chol. (JPG) [file pone.0201373.s002.jpg]

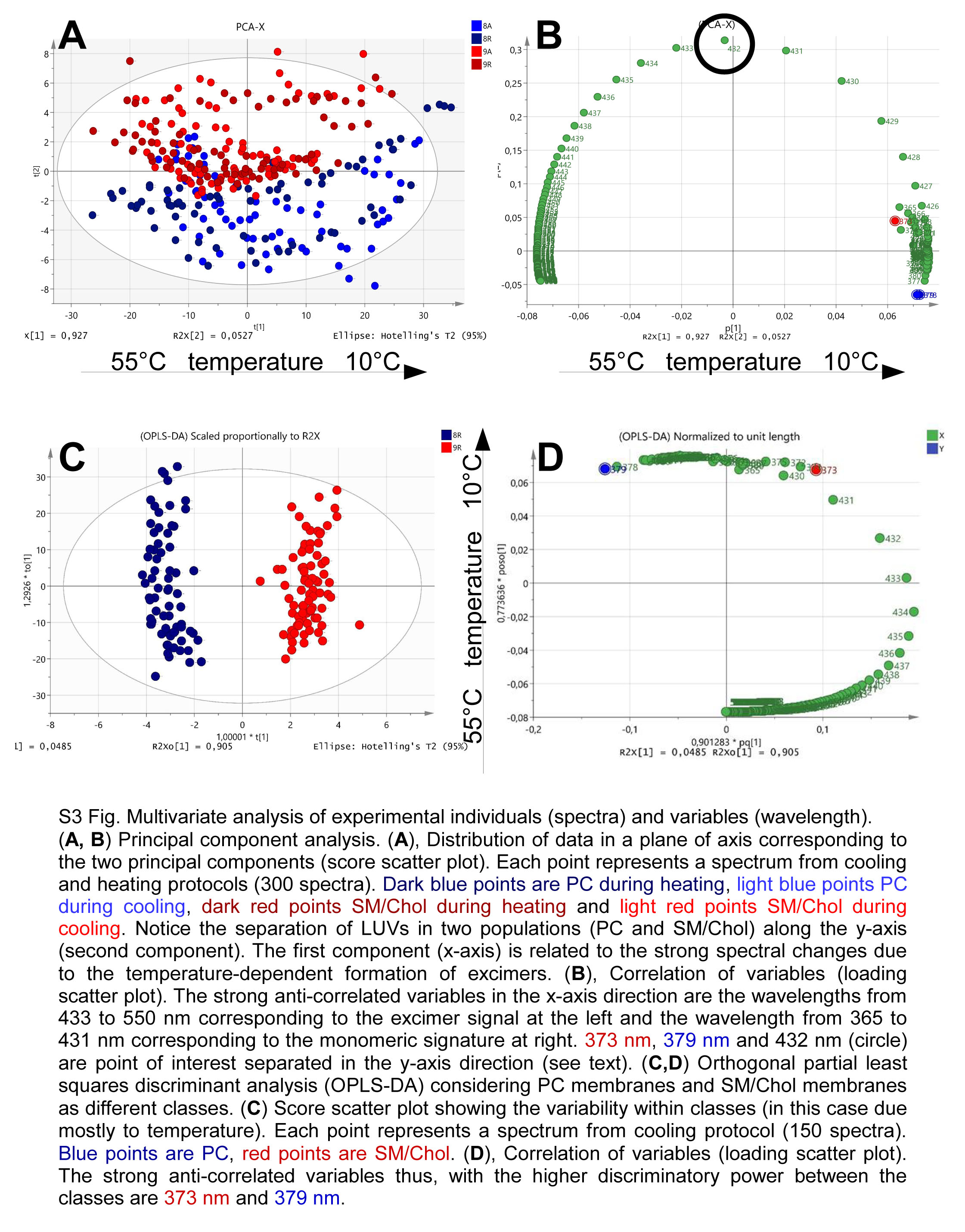

Supplement: S3 Fig — (A, B) Principal component analysis. (A), Distribution of data in a plane of axis corresponding to the two principal components (score scatter plot). Each point represents a spectrum from cooling and heating protocols (300 spectra). In dark blue points PC during heating, light blue PC during cooling, dark red SM/Chol during heating and light red SM/Chol during cooling. Notice the separation of data in two populations (PC and SM/Chol) along the y-axis (second component). The first component (x-axis) is related to the strong spectral changes due to the temperature-dependent formation of excimers. (B), Correlation of variables (loading scatter plot). The strong anti-correlated variables in the x-axis direction are the wavelengths from 433 to 550 nm corresponding to the excimer signal at the left and the wavelength from 365 to 431 nm corresponding to the monomeric signature at right. 373 nm (red circle), 379 nm and 432 nm (blue circle) are points of interest separated in the y-axis direction (see text). (C,D) Orthogonal partial least squares discriminant analysis (OPLS-DA) considering PC and SM/Chol membranes as different classes. (C) Score scatter plot showing the variability within classes (due mostly to temperature). Each point represents a spectrum from cooling protocol (150 spectra). Blue points PC, red SM/Chol. (D), Correlation of variables (loading scatter plot). The strong anti-correlated variables thus, with the higher discriminatory power between the classes are 373 nm and 379 nm. (JPG) [file pone.0201373.s003.jpg]

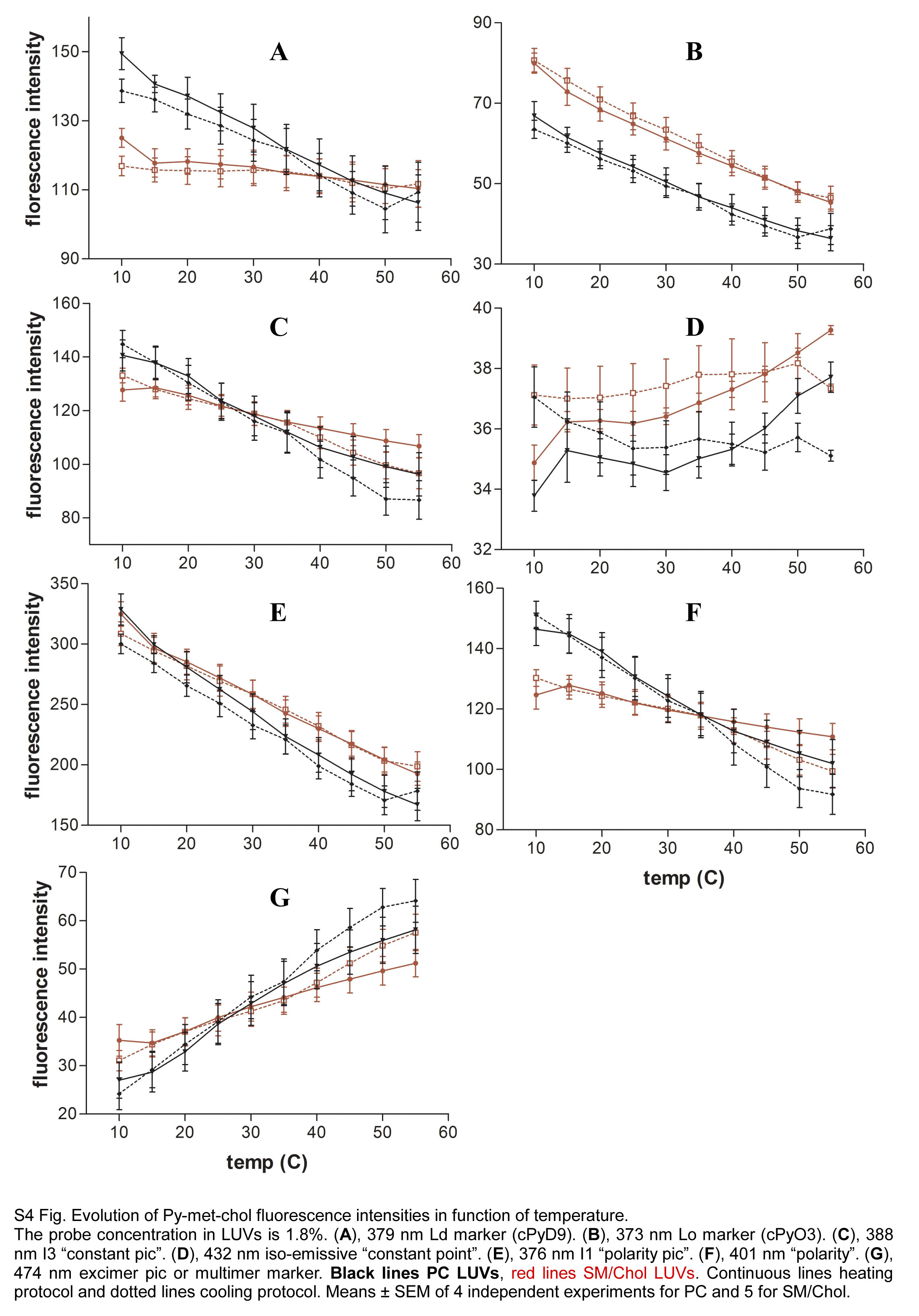

Supplement: S4 Fig — The probe concentration in LUVs is 1.8%. (A), 379 nm Ld marker (cPyD9). (B), 373 nm Lo marker (cPyO3). (C), 388 nm I3 “constant pic”. (D), 432 nm iso-emissive “constant point”. (E), 376 nm I1 “polarity pic”. (F), 401 nm “polarity”. (G), 474 nm excimer pic or multimer marker. Black lines PC LUVs, red lines SM/Chol. Continuous lines heating protocol and dotted lines cooling protocol. Means ± SEM of 4 independent experiments for PC and 5 for SM/Chol. (JPG) [file pone.0201373.s004.jpg]

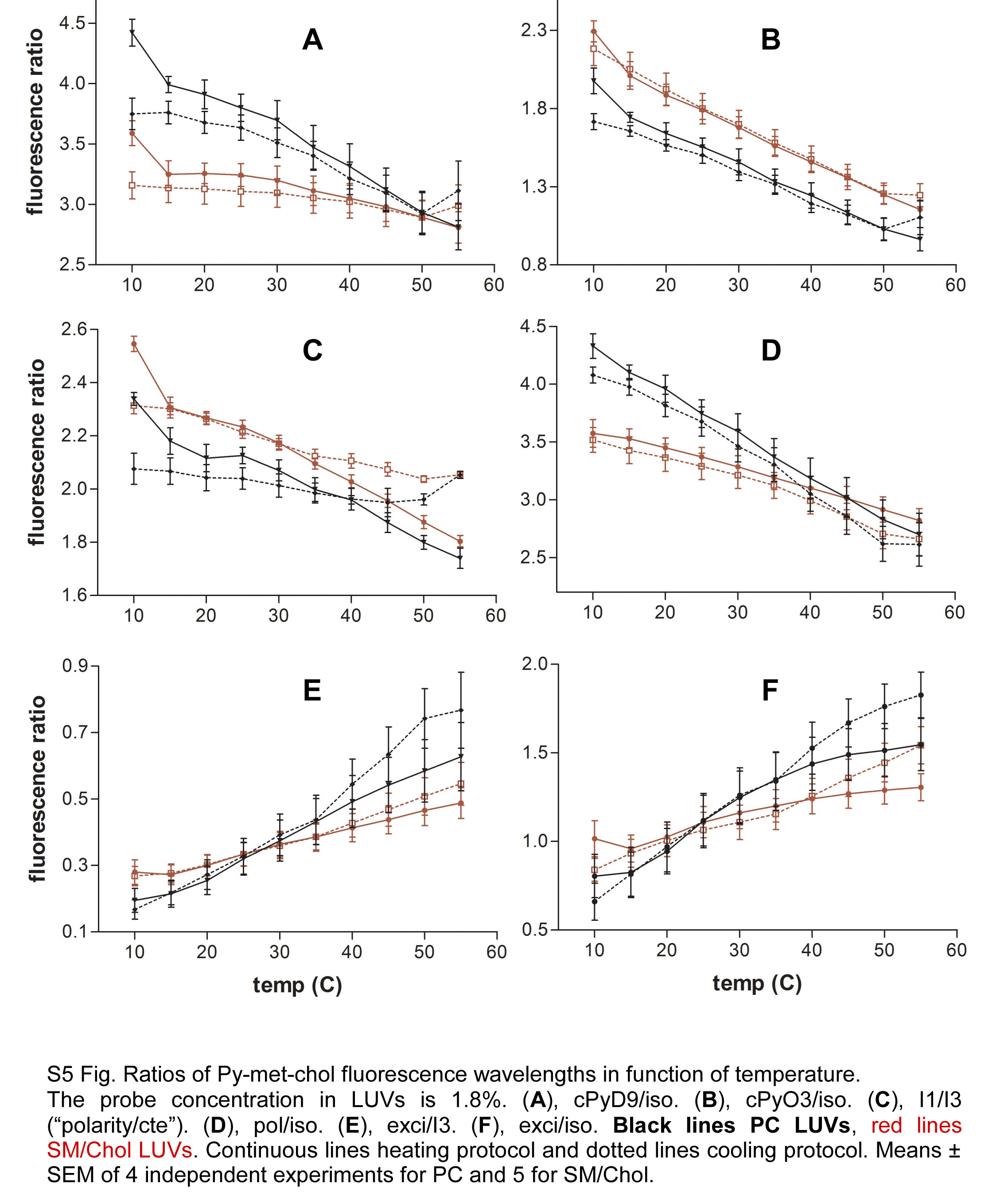

Supplement: S5 Fig — The probe concentration in LUVs is 1.8%. (A), cPyD9/iso. (B), cPyO3/iso. (C), I1/I3 (“polarity/constant”). (D), pol/iso. (E), exci/I3. (F), exci/iso. Black lines PC LUVs, red lines SM/Chol. Continuous lines heating protocol and dotted lines cooling protocol. Means ± SEM of 4 independent experiments for PC and 5 for SM/Chol. (JPG) [file pone.0201373.s005.jpg]

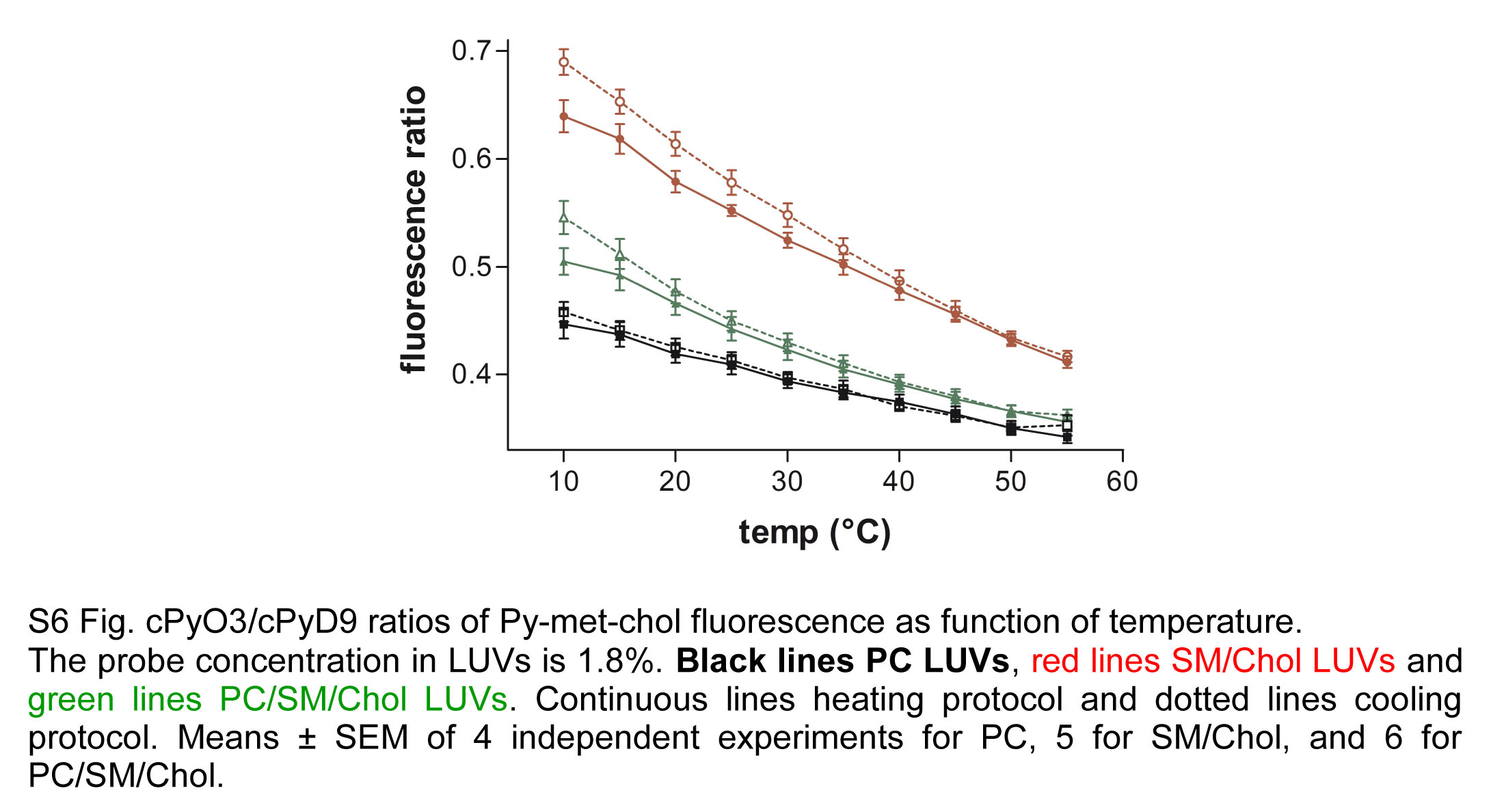

Supplement: S6 Fig — The probe concentration in LUVs is 1.8%. Black lines PC LUVs, red lines SM/Chol and green lines PC/SM/Chol. Continuous lines heating protocol and dotted lines cooling protocol. Means ± SEM of 4 independent experiments for PC, 5 for SM/Chol, and 6 for PC/SM/Chol. (JPG) [file pone.0201373.s006.jpg]

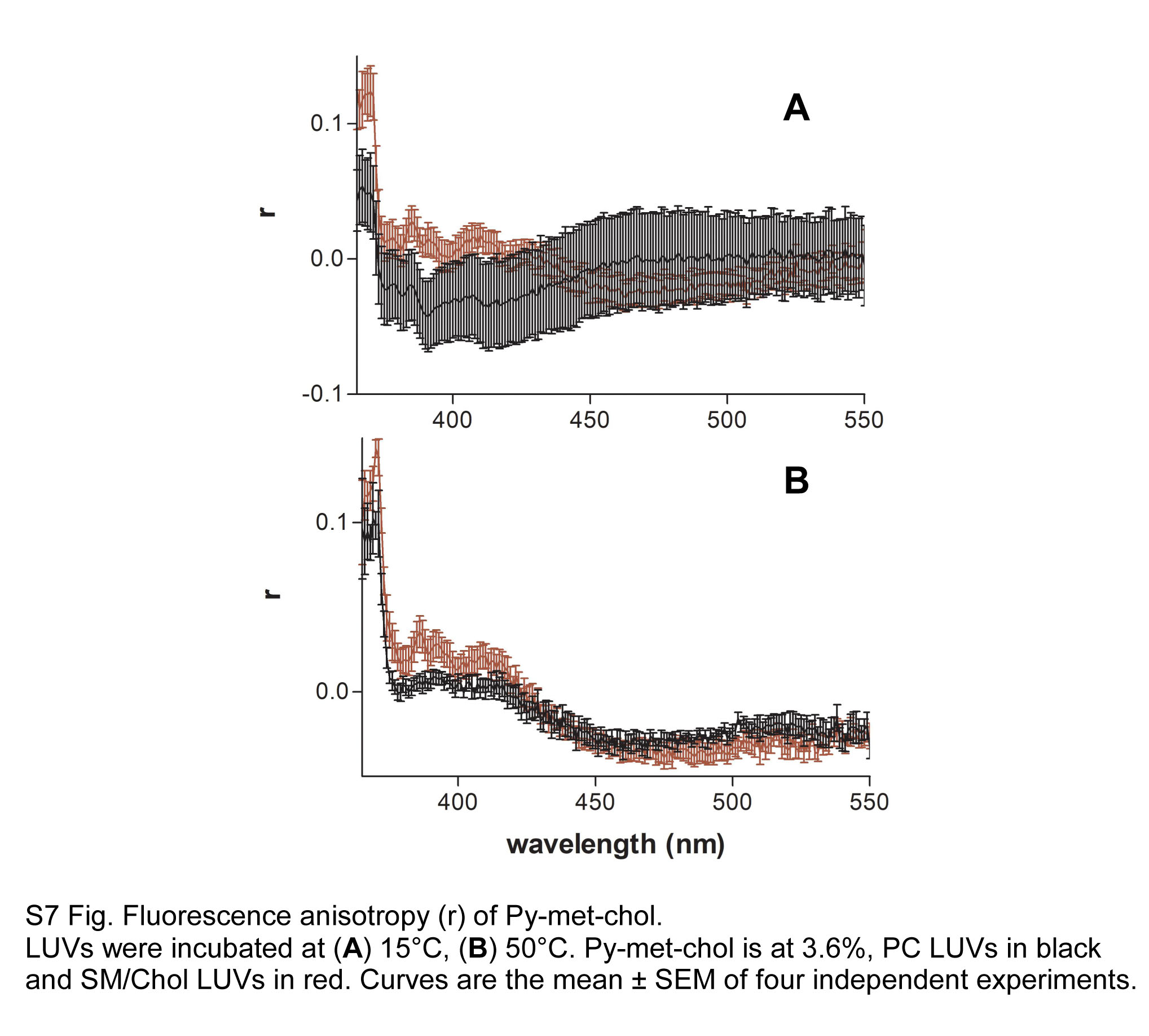

Supplement: S7 Fig — LUVs were incubated at (A) 15°C, (B) 50°C. Py-met-chol is at 3.6%, PC LUVs in black and SM/Chol in red. Curves are the mean ± SEM of four independent experiments. (JPG) [file pone.0201373.s007.jpg]

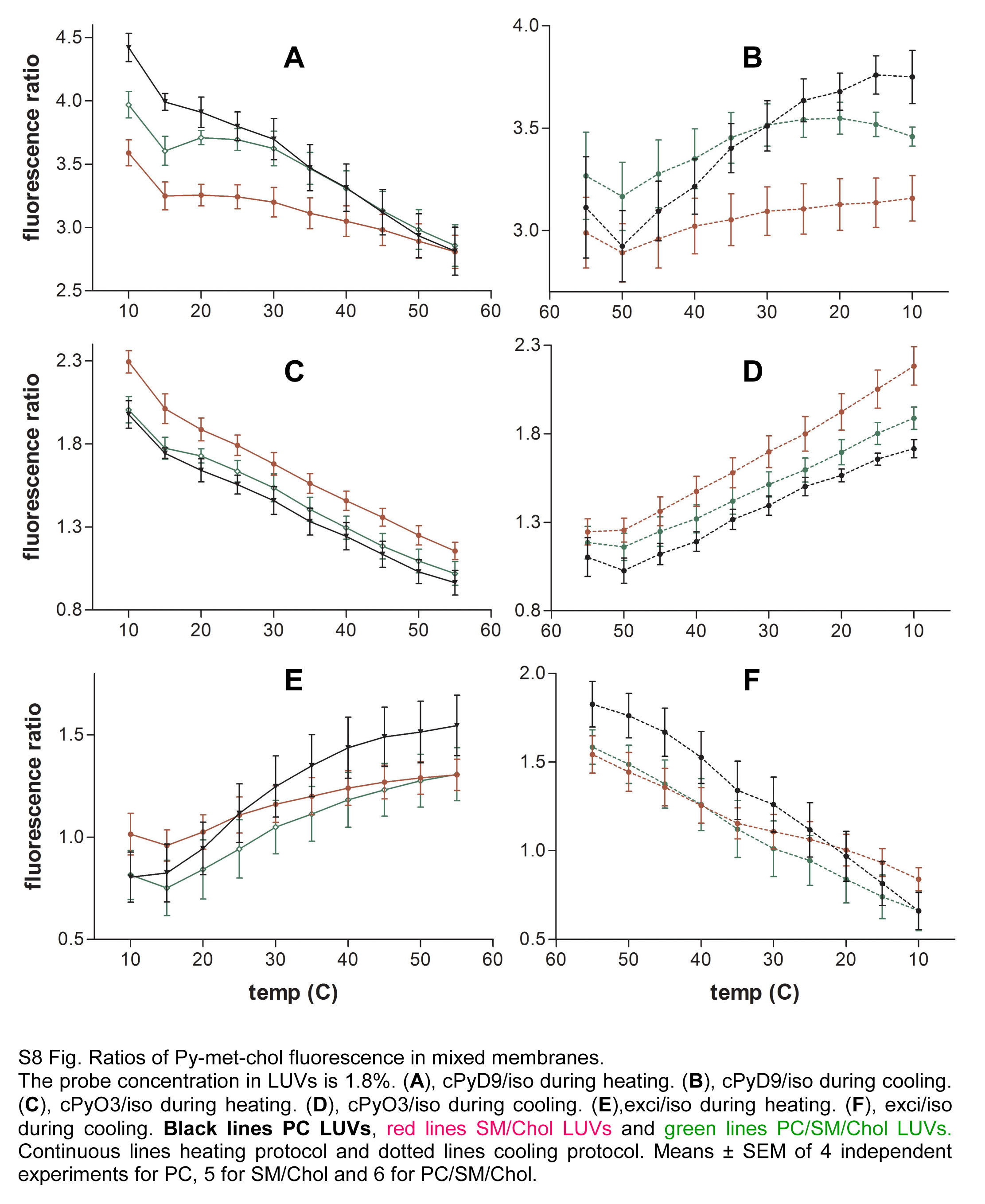

Supplement: S8 Fig — The probe concentration in LUVs is 1.8%. (A), cPyD9/iso during heating. (B), cPyD9/iso during cooling. (C), cPyO3/iso during heating. (D), cPyO3/iso during cooling. (E),exci/iso during heating. (F), exci/iso during cooling. Black lines PC LUVs, red lines SM/Chol and green lines PC/SM/Chol. Continuous lines heating protocol and dotted lines cooling protocol. Means ± SEM of 4 independent experiments for PC, 5 for SM/Chol and 6 for PC/SM/Chol. (JPG) [file pone.0201373.s008.jpg]

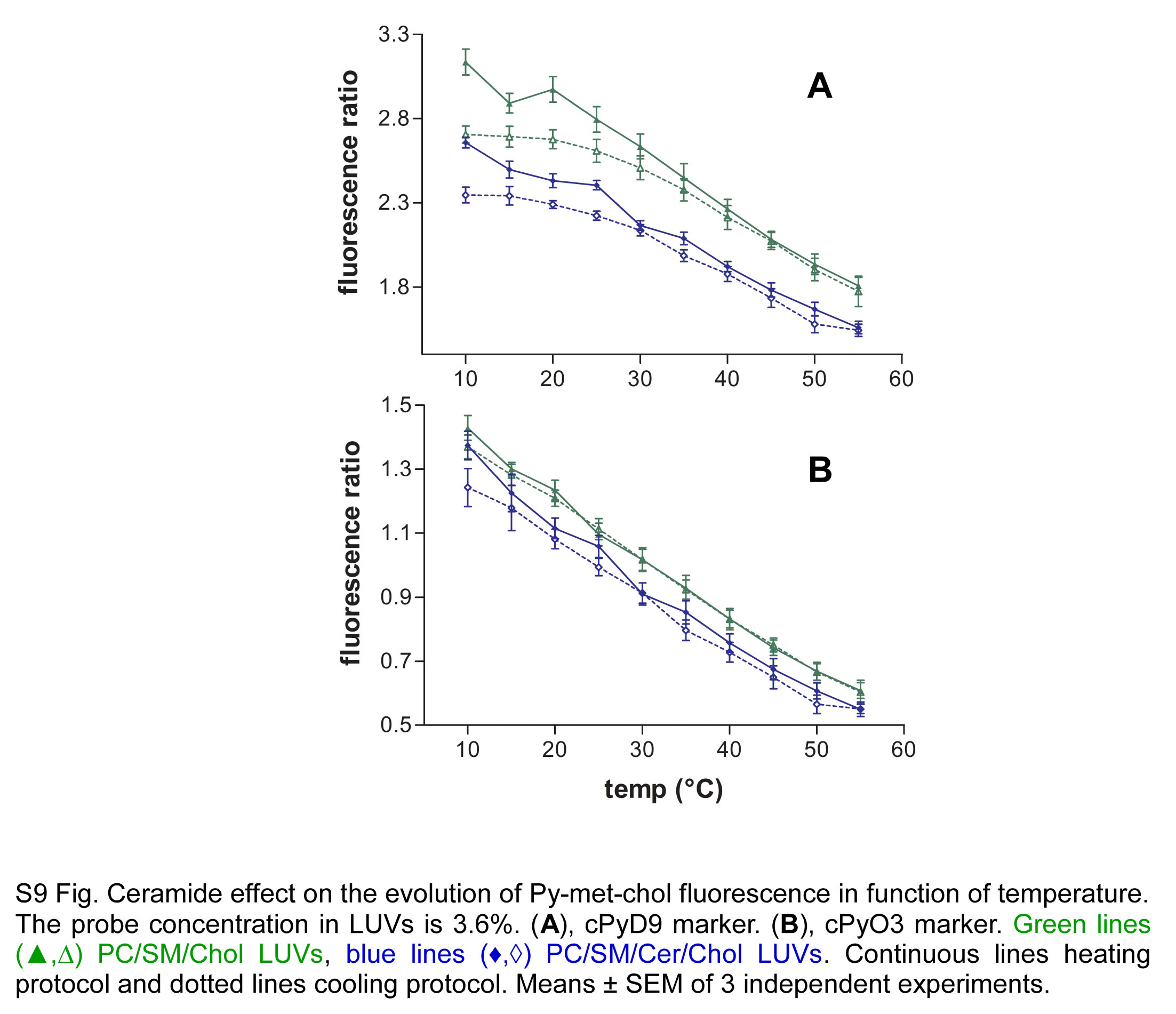

Supplement: S9 Fig — The probe concentration in LUVs is 3.6%. (A), cPyD9 marker. (B), cPyO3 marker. Green lines (▲,∆) PC/SM/Chol LUVs, blue lines (♦,◊) PC/SM/Cer/Chol LUVs. Continuous lines heating protocol and dotted lines cooling protocol. Means ± SEM of 3 independent experiments. (JPG) [file pone.0201373.s009.jpg]
